# Identification and Characterization of Bacterial Diterpene Cyclases that Synthesize the Cembrane Skeleton

**DOI:** 10.1002/cbic.201200651

**Published:** 2013-02-06

**Authors:** Ayuko Meguro, Takeo Tomita, Makoto Nishiyama, Tomohisa Kuzuyama

**Affiliations:** [a] Biotechnology Research CenterThe University of Tokyo 1-1-1 Yayoi, Bunkyo-ku, Tokyo 113-8657 (Japan)

**Keywords:** biosynthesis, cyclization, reaction mechanisms, *Streptomyces*, terpenoids

Terpenoids, which are also known as isoprenoids, are a large and highly diverse group of natural products.^[^^1^^–^^3^^]^ The structural diversity of terpenoids is a result of the cyclization of a limited number of linear polyprenyl diphosphate substrates, such as geranyl diphosphate (GDP, C10), farnesyl diphosphate (FDP, C15), and geranylgeranyl diphosphate (GGDP, C20), to generate monocyclic or multicyclic compounds. Terpene cyclases play a key role in the biosynthetic processes of terpenoids, because they yield structurally and stereochemically diverse ring skeletons in the reaction cascade, which involves ionization, hydride shifts, deprotonation, protonation, methyl migration, and hydroxylation.^[^^1^^,^^3^^]^ The terpene cyclase reaction cascade is initiated by the formation of a highly reactive carbocation. There are two mechanisms for this carbocation formation: 1) ionization of the polyprenyl diphosphate substrate by diphosphate abstraction, and 2) protonation of the terminal olefin of the substrate.^[^^3^^–^^7^^]^ Terpene cyclases are classified according to their mechanisms.^[^^3^^–^^7^^]^ Class I terpene synthases initiate carbocation formation by substrate ionization, whereas class II terpene cyclases initiate carbocation formation by substrate protonation. Class I terpene synthases contain DDXXD/E and/or NSE/DTE motifs, which chelate the Mg^2+^ ions that are used to bind the diphosphate moiety of the substrate and are required for the ionization of the allylic diphosphate ester bond. The class II terpene cyclases, in contrast, possess a DXDD motif, which serves as the catalytic acid that protonates the terminal carbon–carbon double-bond of the substrate. Note that a diphosphate group is missing in the reaction product that is formed by class I enzymes, whereas a diphosphate group is retained in the reaction product formed by class II enzymes.

Members of the *Streptomyces* genus and other actinomycetes, which are Gram-positive bacteria, are known to produce an enormous variety of natural products. However, few diterpenes (C20) have been isolated from these bacteria. A limited number of diterpene cyclases that react with GGDP having been cloned from these bacteria and characterized, for example, terpentedienyl diphosphate synthase (Cyc1) from terpentecin-producing *Kitasatospora griseola*,^[^^8^^,^^9^^]^ *ent*-copalyl diphosphate synthase (SsCPS) from viguiepinol-producing *Streptomyces* sp. KO-3988,^[^^10^^,^^11^^]^ halimadienyl diphosphate synthase (Rv3377c) from *Mycobacterium tuberculosis* H37,[12] and cyclooctat-9-en-7-ol synthase (CotB2) from cyclooctatin-producing *Streptomyces melanosporofaciens* MI614-43F2[13] (Figure S1 in the Supporting Information). Of these four enzymes, Cyc1, SsCPS, and Rv3377c are considered to be class II terpene cyclases. As mentioned above, a diphosphate group is found in the reaction product formed by these class II enzymes. These class II enzymes are accompanied by class I terpene synthases Cyc2,[8] ORF3,[10] and Rv3378c,[14] which react with each reaction product of the class II terpene cyclases (Cyc1, SsCPS, and Rv3377c, respectively) to release a diphosphate group (Figure S1). Interestingly, Cyc1 and SsCPS were found by their proximity to the mevalonate pathway gene cluster, which provides isoprene unit precursors of GGDP.^[^^8^^,^^10^^]^ However, of the four above-mentioned enzymes, CotB2 is considered to be a class I diterpene synthase because it possesses an NSE motif.[13] In addition, a diphosphate group is removed by the reaction catalyzed by CotB2 to form the reaction product cyclooctat-9-en-7-ol.

Here, we report two novel diterpene cyclases, DtcycA and DtcycB, that were mined from *Streptomyces* sp. SANK 60404, which was not previously known to be a terpenoid producer.^[^^15^^,^^16^^]^ To mine these diterpene cyclases from the bacterium, we directed our attention to genes in close proximity to a gene encoding a GGDP synthase, which is indispensable for diterpene production. There are two reasons for this choice of approach: 1) the bacterial diterpene cyclases mentioned above exhibit only a low level of overall sequence similarity, whereas the GGDP synthases from these bacteria display more than 30 % identity,^[^^8^^,^^10^^,^^13^^]^ and 2) a GGDP synthase gene is located near a diterpene cyclase gene in the genomes of these bacteria (Figure S2).

We first de novo sequenced the genome of *Streptomyces* sp. SANK 60404. An assembly of the sequence reads (estimated coverage, 124-fold) yielded 1565 contigs with a total of 7 706 959 base pairs. To mine the unidentified diterpene cyclases from this bacterium, we first attempted to retrieve the GGDP synthases in the draft sequence because biochemically characterized diterpene cyclases are frequently located in close proximity to GGDP synthase genes, as mentioned above. Using the BLAST program with the amino acid sequence of CotB1 (a *Streptomyces* GGDP synthase for cyclooctatin biosynthesis)[13] as the query, we found five contigs containing open reading frames that displayed 31–32 % sequence identity to CotB1. We then searched the regions flanking these five open reading frames for putative terpene synthases that include DDXXD/E and/or NSE/DTE motifs (class I terpene synthases) or DXDD motif (class II terpene cyclases). We found two genes that encoded putative class I terpene synthases (DtcycA and DtcycB) in the regions that flank the GGDP synthase genes, but no gene encoding a putative class II terpene cyclase (Figure S3). DtcycA (371 amino acids, DDBJ/EMBL/GenBank accession number AB738084) has a clear NSE motif, but exhibits only 14 % identity to CotB2 over 332 amino acids. DtcycB (343 amino acids, accession number AB738085) has a clear NSE motif, but exhibits only 19 % identity to CotB2 over 308 amino acids (Figure S4).

With DtcycA and DtcycB as the query sequences, we then performed BLAST searches of the NCBI protein database, and identified terpene cyclase homologues (*E* values<1×10^−10^). For the closest homologues, DtcycA showed 27 % identity over 369 amino acids with a putative terpene cyclase (Protein ID, YP_004922572) from *Streptomyces flavogriseus* ATCC 33331, and DtcycB showed 23 % identity over 338 amino acids with a putative terpene cyclase (Protein ID, ZP_06911744) from *Streptomyces pristinaespiralis* ATCC 25486. Both homologues are found in a phylogenetic dendrogram based on the analysis of amino acid sequences from bacterial sesquiterpene (C15) cyclase homologues.[17] In addition, it has been reported that sesquiterpene cyclases with similar sequences produce the same major sesquiterpene.[17] Therefore, if a highly homologous terpene cyclase has previously been characterized, a phylogenetic analysis of terpene cyclases could allow prediction of their reaction products. However, we were unable to predict the reaction products synthesized by DtcycA and DtcycB, because none of the closely homologous terpene cyclases has been characterized.

Therefore, to elucidate the enzymatic functions of DtcycA and DtcycB, we overexpressed the corresponding genes in *Escherichia coli* and characterized the gene products. The molecular masses of the recombinant DtcycA and DtcycB enzymes were estimated at 42 and 38 kDa, respectively, by SDS-PAGE, and at 95 and 69 kDa, respectively, by gel filtration chromatography. These results suggest that both proteins are likely homodimers (Figures S5 and S6). A functional analysis of the recombinant enzymes when using GDP (C10), FDP (C15), and GGDP (C20) as substrates indicated that only GGDP was converted into several reaction products in the presence of MgCl_2_. The two main products formed by the DtcycA reaction were detected at 10:29 and 11:13 min in the GC analysis, whereas the three main products formed by DtcycB were detected at 10:04, 11:11, and 11:13 min (Figures 1 and S7). In the absence of MgCl_2_, no products were detected in the reaction mixtures.

**Figure 1:**
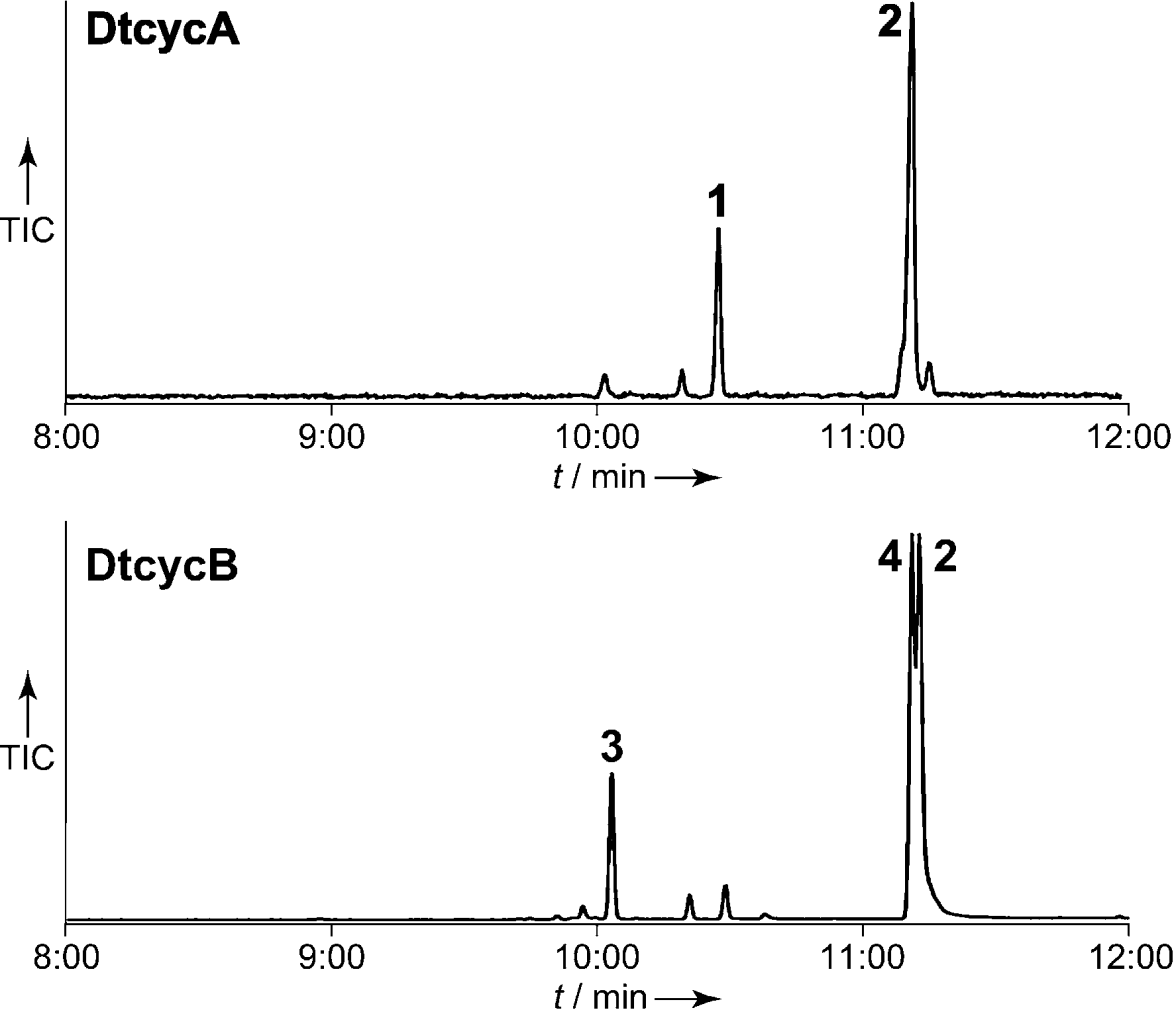
GC-MS analysis of the diterpene products. Two diterpenes (1 and 2) were generated by the DtcycA-catalyzed reaction, and three diterpenes (2, 3, and 4) were generated by the DtcycB-catalyzed reaction. The retention times of 1, 2, 3, 4, and the geranylgeraniol standard were 10:29, 11:13, 10:04, 11:11, and 11:31 min, respectively. The MS spectra of diterpene products 1, 2, 3, and 4 are presented in Figure S7. TIC, total ion current.

Large-scale preparation of the reaction products allowed us to deduce the structures of these compounds. The two DtcycA-catalyzed reaction products were determined to be the isopropylidene isomer of cembrene C (**1**, (1*E*,5*E*,9*E*)-1,5,9-trimethyl-12-(propan-2-ylidene)cyclotetradeca-1,5,9-triene)^[^^18^^,^^19^^]^ and (*R*)-nephthenol (**2**);[20] two of the three DtcycB-catalyzed reaction products were identified as (*R*)-nephthenol (**2**) and (*R*)-cembrene A (**3**).^[^^18^^,^^20^^]^ These structures, with the exception of **1**, were elucidated by comparison of the ^1^H and ^13^C NMR spectral data and the specific optical rotation of each product with literature data (Tables S1 to S3). Although **1** is a known compound (the isopropylidene isomer of cembrene C), we determined the structure of **1** by extensive NMR spectroscopic analysis, because of the lack of spectral data for the isopropylidene isomer of cembrene C in the literature (Figure S8–S12).^[^^18^^,^^19^^]^ We thus conclude that both DtcycA and DtcycB encode diterpene cyclases that are capable of forming multiple diterpene products.

We were unable to find the proposed structure of **4** in the Dictionary of Natural Products on DVD ver. 21:1 (CRC Press) or in the SciFinder database (American Chemical Society). The molecular formula of **4** was deduced to be C_20_H_34_O by positive high-resolution mass spectrometry (*m*/*z* 291.2690 [*M*+H]^+^; calculated for C_20_H_35_O: 291.2688). The ^1^H NMR spectrum (Figure S13) indicated the presence of three olefinic protons (*δ*=4.94, 5.05, and 5.16 ppm; each 1 H, triplet, *J*=6.0, 5.9, and 7.8 Hz). In addition, three tertiary olefinic carbon signals (*δ*=120.8, 124.4, 125.5 ppm), three quaternary olefinic carbon signals (*δ*=134.0, 134.2, and 135.3 ppm), and one quaternary paraffinic carbon signal (*δ*=38.0 ppm) were apparent in the ^13^C NMR spectrum of **4** (Figure S14). These results indicate that compound **4** is a monocyclic compound with three double bonds in the ring. An oxygen-bearing methine signal (^1^H, *δ*=3.35 ppm; doublet, *J*=9.5 Hz; ^13^C, 72.6 ppm) was also observed, which indicates the presence of a hydroxyl group in **4**. Of the five methyl groups, two belong to an isopropyl group (^1^H, *δ*=0.83 and 0.87; each 3 H, singlet; ^13^C, *δ*=24.0 and 22.9 ppm, respectively). The remaining methyl groups exhibited resonances that are typical of allylic methyl groups (^1^H, *δ*=1.54, 1.58 and 1.61; each 3 H, singlet; ^13^C, *δ*=16.1, 16.0 and 16.5 ppm, respectively). The 14 remaining protons appeared in the *δ*=1.55–2.30 ppm region (Table 1). The directly bonded carbon and hydrogen atoms were assigned based on the HSQC spectrum (Figure S15). By using extensive NMR spectroscopic analysis, which included COSY and HMBC experiments (Figures S16 and S17), we determined that the planar structure of **4** is (4*E*,8*E*,12*E*)-2,2,5,9,13-pentamethylcyclopentadeca-4,8,12-trien-1-ol (Scheme 1).

**Table 1:** ^1^H and ^13^C NMR spectral data for diterpene 4.

		*δ*_C_	*δ*_H_ (*J* [Hz])	HMBC[a]
1	CH-O	72.6	3.35, d (9.5)	2, 14, 15, 16
2	C	38.0		
3	CH_2_	39.0	2.30, dd (14.2, 9.4); 1.68, dd (14.2, 6.2)	1, 4, 5, 17
4	CH=	120.8	5.16, t (7.8)	18
5	C=	135.3		
6	CH_2_	38.7	2.11, m; 2.07, m	8
7	CH_2_	24.4	2.22, t (3.5); 2.16, m	6, 8, 9
8	CH=	124.4	4.94, t (6.0)	6, 7, 10
9	C=	134.2		
10	CH_2_	39.2	2.07, t (6.9)	9, 12
11	CH_2_	24.7	2.16, m; 2.07, t (6.9)	9, 12
12	CH=	125.5	5.05, t (5.9)	11, 14, 20
13	C=	134.0		
14	CH_2_	35.1	2.19, m; 1.99, m	1, 12, 15, 20
15	CH_2_	27.4	1.55, m; 1.28, m	1
16	CH_3_	24.0	0.83, s	1, 3, 17
17	CH_3_	22.9	0.87, s	3, 16
18	CH_3_	16.5	1.61, s	4, 5, 6
19	CH_3_	16.1	1.54, s	8, 9, 10
20	CH_3_	16.0	1.58, s	12, 13, 14

The data were recorded in CDCl_3_.

[a] Proton showing HMBC correlation to indicated carbon.

**Scheme 1:**
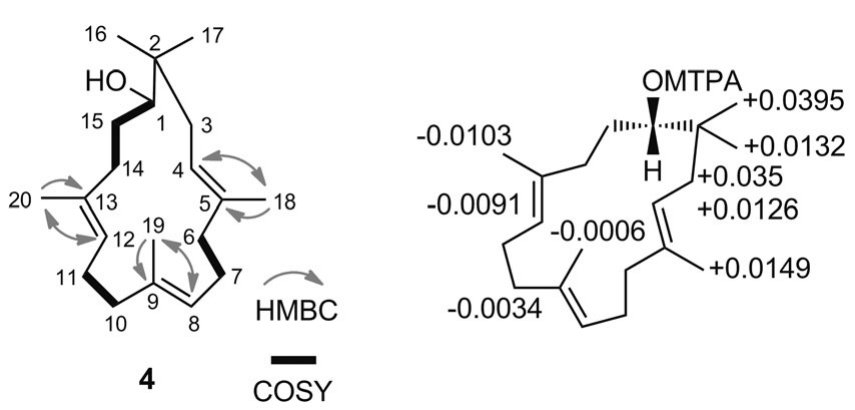
Structure of novel diterpene 4. Left: selected key HMBC and COSY correlations of 4. Right: determination of the absolute stereochemistry of 4 by the modified Mosher method. The values (in ppm) of Δ*δ*=*δ*(*S*)−*δ*(*R*) for the (*S*,*R*)-MTPA derivatives of 4 are presented in the structure. The values of both *δ*(*S*) and *δ*(*R*) for the (*S*,*R*)-MTPA derivatives are described in Figure S18.

The absolute stereochemistry of **4** was assigned by using the modified Mosher method.[21] The reaction of compound **4** with (*R*,*S*)-α-methoxy-α-(trifluoromethyl)phenylacetyl (MTPA) chloride in pyridine formed (*S*,*R*)-MTPA esters of **4**. Based on the ^1^H-shifting values (Δ*δ*=*δ*(*S*)−*δ*(*R*)) of the (*S*,*R*)-MTPA derivatives, the absolute configuration of the secondary alcohol of **4** was determined to be *S* (Scheme 1 and Figure S18). Thus, the absolute stereochemistry of **4** was determined to be *S*.

None of the products formed by DtcycA and DtcycB possessed a diphosphate group, which suggests that the DtcycA and DtcycB enzymes initiate carbocation formation with substrate ionization. Therefore, we propose an ionization-dependent cyclization reaction mechanism for the multiple products that are formed by DtcycA and DtcycB (Scheme 2). The mechanism for the reaction catalyzed by the DtcycA enzyme presumably involves ionization of GGDP and formation of a cyclopropyl carbocation intermediate (**5**). Deprotonation of **5** at C-14 yields **1**, and the subsequent attack at the C-15 position of **5** by a water nucleophile yields compound **2**. The mechanism of the reaction catalyzed by the DtcycB enzyme also involves the ionization of GGDP and the formation of **5**. Compound **2** is then formed as in the mechanism catalyzed by DtcycA. In addition, the deprotonation of **5** at C-16 yields **3**, and the nucleophilic attack at the C-14 position of **5** by water yields the novel compound **4**.

**Scheme 2:**
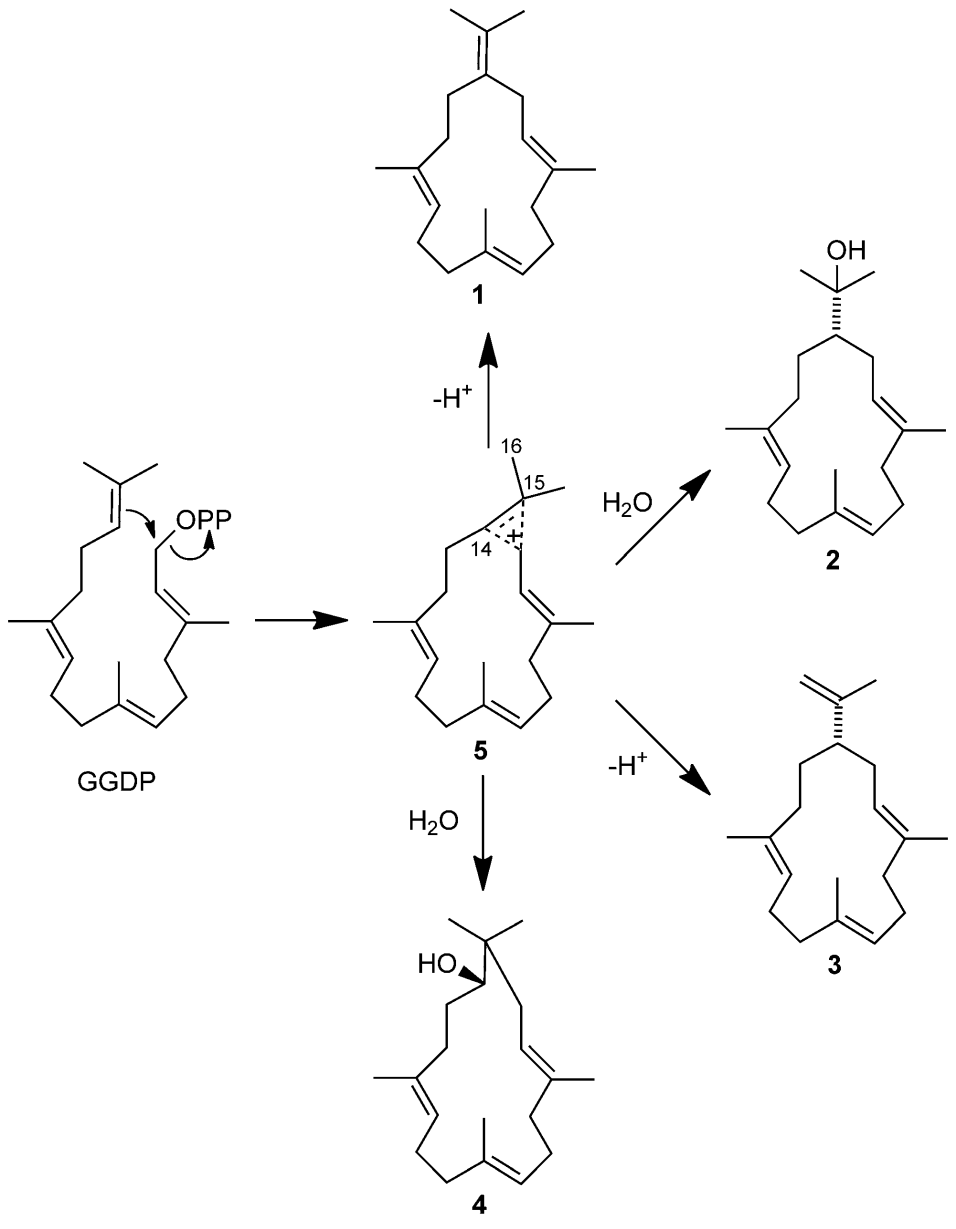
Proposed reactions mechanisms for the formation of DtcycA and DtcycB reaction products.

We calculated the steady-state kinetic constants of DtcycA and DtcycB when using GGDP as the substrate. A typical hyperbolic curve was obtained for product formation as a function of substrate concentration (GGDP, Figure S5 and S6), which indicates that the reaction exhibits Michaelis–Menten kinetics. The steady-state kinetic constants for each cyclase were estimated by incubating the reactions for 3 min and using various GGDP concentrations (10–200 μm). This process yielded a *K*_m_ value of 93.7±8.4 μM and a *k*_cat_ value of 2.8 min^−1^ for the recombinant DtcycA construct, and *K*_m_ 42.1±7.3 μM and *k*_cat_ 1.3 min^−1^ for recombinant DtcycB; these values are comparable to those obtained for the previously characterized Cyc1 from *Kitasatospora griseola*,[9] SsCPS from *Streptomyces* sp. KO-3988,[11] and Rv3377c from *Mycobacterium tuberculosis* H37 (Table 2).[12]

**Table 2:** Steady-state kinetic parameters of the diterpene cyclases.

Diterpene cyclase	*K*_m_ [μM]	*k*_cat_ [min^−1^]	Ref.
DtcycA	93.7±8.4[a]	2.8	this study
DtcycB	42.1±7.3[a]	1.3	this study
Cyc1	64.2±5.7	5.13	[9]
SsCPS	13.7±1.0	1.98	[10]
Rv3377c	11.7±1.9	12.7	[12]

[a] Values are expressed as the mean±SD of three independent experiments.

Finally, to determine whether diterpenes **1**, **2**, **3**, and **4** are produced by *Streptomyces* sp. SANK 60404 in vivo, we attempted to detect these diterpenes in the culture broths of the strain cultivated in seven different types of liquid media and on solid medium. However, GC-MS analysis revealed no production of **1**, **2**, **3**, or **4** in the culture broths under any of the culture conditions used in this study, which suggests that the genes encoding the DtcycA and DtcycB enzymes are not expressed in *Streptomyces* sp. SANK 60404 under these standard laboratory culture conditions. However, it is also possible that diterpenes **1**, **2**, **3**, and **4** are converted into as-yet unidentified products by *Streptomyces* sp. SANK 60404. Interestingly, the DtcycA and DtcycB genes are flanked by hypothetical genes that might be involved in the modification of these diterpene products (Figure S3).

In this study, we found two genes that encode diterpene cyclases, DtcycA and DtcycB, in proximity to GGDP synthase genes in the genome of *Streptomyces* sp. SANK 60404. In contrast, BLAST searches with CotB2 as the query sequence failed to find DtcycA or DtcycB in the draft genome sequence of *Streptomyces* sp. SANK 60404. This finding suggests that database searches that are based on proximity to GGDP synthase genes can be used to find novel diterpene cyclases in public protein databases, and that many currently unrecognized diterpene cyclases might be buried in public protein databases. We then identified diterpene products synthesized by recombinant DtcycA and DtcycB. Serendipitously, these diterpene cyclases formed a diterpene with a novel skeleton and three cembrane diterpenes that have previously been isolated from soft coral^[^^18^–^20^^]^ and some plants,^[^^22^^,^^23^^]^ but not from bacteria. However, DtcycA and DtcycB show no sequence similarity to cembrene A synthase (CAS2, 614 amino acids, accession number XM_002513288) from the castor oil plant (*Ricinus communis*)[22] or to cembratrien-ol synthases (NsCBTS2a, 598 amino acids, accession number HM241151; NsCBTS2b, 598 amino acids, accession number HM241152; NsCBTS3, 598 amino acids, accession number HM241153) from tobacco (*Nicotiana sylvestris*).[23] Thus, DtcycA and DtcycB are novel diterpene cyclases that synthesize the cembrane skeleton.

The crystal structures of two plant diterpene cyclases have been previously resolved: taxadiene synthase and *ent*-copalyl diphosphate synthase.^[^^24^^,^^25^^]^ These plant enzymes have three α-helical domains (α, β, and γ). The active site of taxadiene synthase (a class I synthase) is located at the α domain, and an aspartate-rich motif and an NSE/DTE motif are found on opposite sides of the entrance to the active site pocket. In class I synthase family members, these motifs are involved in the binding of Mg^2+^ ions, which is indispensable for substrate recognition. Because DtcycA and DtcycB catalyze the Mg^2+^-dependent cyclization of GGDP, these motifs were expected to be conserved in both enzymes. In CotB2, which catalyzes the Mg^2+^-dependent cyclization of GGDP, both motifs were clearly detected. However, the alignment of DtcycA and DtcycB with other bacterial class I diterpene synthases clearly revealed NSE/DTE motifs, but the presence of aspartate-rich motifs was ambiguous (Figure S4). A previous study reported that only the NSE/DTE motif is frequently conserved in class I terpene synthases.[26] Further insights into the structural basis of substrate recognition require the determination of the crystal structures of the DtcycA and DtcycB enzymes complexed with their substrates and Mg^2+^ ions. Such crystal structures might be able to answer the particularly intriguing question of why both DtcycA and DtcycB yield **2** in spite of their low sequence similarity.

## Experimental Section

**Reagents, bacterial strains, and vectors:** *Streptomyces* sp. SANK 60404 was a gift from Daiichi Sankyo (Tokyo, Japan), which had previously isolated the strain from a soil sample collected in Okinawa, Japan.^[^^15^^,^^16^^]^ *E. coli* DH5α and *E. coli* BL21(DE3) (Takara Bio, Tokyo, Japan) were used for plasmid cloning and recombinant protein expression, respectively. The pT7Blue T-Vector (Novagen/Merck Millipore) and the pHis8 vector[27] were used to clone PCR products and express proteins, respectively. GGDP was synthesized as previously described.[28]

**Genome sequencing of** ***Streptomyces*** **sp. SANK 60404:** The genomic DNA of *Streptomyces* sp. SANK 60404 was purified[29] and sequenced by using the Genome Analyzer IIx system (Illumina, San Diego, CA) at Takara Bio, Kyoto. The sequence reads were assembled by using the Edena de novo short-reads assembler (Genomic Research Laboratory, Geneva, Switzerland).[30] Genome sequences were annotated by using the programs MiGAP (http://www.migap.org/index.php/en), protein BLAST (http://blast.ncbi.nlm.nih.gov/Blast.cgi),[31] and FramePlot 2.3.2 (2002) (http://www0.nih.go.jp/∼jun/cgi-bin/frameplot.pl).[32]

**Protein expression and purification:** PCR amplification from *Streptomyces* sp. SANK 60404 genomic DNA was used to generate a DNA fragment. Ligation into the *E. coli* expression vector pHis8 yielded the pHIS8cyc1 construct (containing DtcycA). The reaction was performed with forward primer 5′-GGGGG AATTC ATGAC AGACC CAGCC GTGAC (EcoRI site underlined) and reverse primer 5′-GGGGA AGCTT TCACT GGTCG AGTTG TTCCC (HindIII site underlined). The same protocol was used for the construction of pHIS8cyc2 (DtcycB) but with the following primers: forward primer 5′-GGGGG AATTC ATGGA TCTTC CTCCC GCCC (EcoRI site underlined) and reverse primer 5′-GGGGA AGCTT TCAGG CCCCA GCCTG CGCCC (HindIII site underlined). The pHIS8cyc1 and pHIS8cyc2 constructs were introduced into *E. coli* BL21(DE3) cells, and transformants were grown in terrific broth (TB) containing kanamycin (50 μg mL^−1^) at 37 °C. When the absorbance at 600 nm reached approximately 1.0, isopropyl-β-D-thiogalactopyranoside (IPTG) was added (final concentration 0.5 mm) to induce protein expression. After of cultivation (12–14 h, 18 °C), cells were harvested by centrifugation (10 000 *g*, 10 min), and suspended in lysis buffer (Tris**⋅**HCl (50 mm, pH 8.0), NaCl (500 mm), imidazole (20 mm), Tween 20 (1 % *v*/*v*), and glycerol (20 % *v*/*w*)). The cell suspension was then sonicated on ice by using a Branson Sonifier 250 (Emerson Japan, Atsugi, Japan). To separate the cellular debris from soluble protein, the lysate was centrifuged (30 000 *g*, 4 °C, 20 min), and the resulting supernatant was loaded onto an Ni-NTA Superflow column (Qiagen). After being washed with a wash buffer (Tris**⋅**HCl (50 mm, pH 8.0), NaCl (500 mm), imidazole (20 mm), and glycerol (20 % *v*/*w*)), the desired protein was eluted with imidazole (250 mm) in the same buffer. The purified His-tagged recombinant protein was dialyzed against dialysis buffer B (Tris**⋅**HCl (50 mm, pH 8.0) and NaCl (100 mm)). All recombinant proteins were concentrated by centrifugation in a Vivaspin (10 000 Da MWCO, 4 °C; Sartorius Stedim Biotech, Göttingen, Germany). The concentrated sample was applied to a HiLoad 26/60 Superdex 75 gel-filtration column (GE Healthcare), which was equilibrated with buffer C (Tris**⋅**HCl (20 mm, pH 8.0) and NaCl (150 mm)), and eluted at 2.5 mL min^−1^. The purity of both enzymes was over 95 % as determined by SDS-PAGE.

**Enzyme assay with polyprenyl diphosphate substrates:** The enzyme assays were performed in HEPES**⋅**NaOH (50 mm, pH 7.5) containing MgCl_2_ (5 mm), polyprenyl diphosphate (1 mm; GDP, FDP, or GGDP), and recombinant enzyme (1 mg mL^−1^). The reaction mixture (500 μL) was incubated at 30 °C for 2 hrs. After incubation, the reaction mixture was extracted with ethyl acetate (500 μL). The organic extracts were then evaporated to dryness under vacuum, and the residues were reconstituted with ethyl acetate (100 μL) for GC-MS analysis (see below).

**GC-MS analysis:** The reaction products were analyzed in a 6890N GC-MS instrument (Agilent Technologies). The sample (1 or 2 μL) was introduced by split injection (230 °C) onto an InertCap 5MS/Sil column (phenyl (5 %), methyl polysilarylene (95 %); 15 m×0.25 mm i.d., 0.25 μm film thickness; GL Sciences, Tokyo, Japan). The column temperature was 70 °C (maintained for 1 min after injection) and then increased (15 °C min^−1^) to 280 °C (maintained for the remainder of the 20 min program).

**Large-scale preparation of diterpene products:** The large-scale production of the diterpene products was performed in a total volume of 350 mL. The reaction was performed in HEPES**⋅**NaOH (50 mm, pH 7.5) containing MgCl_2_ (2 mm), GGDP (0.5 mm), and recombinant enzyme (up to 0.2 mg mL^−1^, added in two consecutive 4 h steps). The reaction mixture was incubated (30 °C, 18 h) and then extracted twice with ethyl acetate (350 mL). After drying over Na_2_SO_4_, the organic layer was evaporated in vacuo, and the residue was dissolved in methanol (1 mL). The reaction products were purified by preparative HPLC with a PEGASIL ODS column (20×250 mm; Senshu Scientific, Tokyo, Japan) and isocratic elution (methanol (90 %), 8 mL min^−1^). The absorption of the eluate was monitored at 210 nm. In a single preparation, **1** (4.0 mg) and **2** (3.0 mg) were obtained from the DtcycA-catalyzed reaction mixtures, and **2** (5.0 mg), **3** (5.4 mg), and **4** (2.4 mg) were obtained from the DtcycB-catalyzed reaction mixtures. To prepare the (*R*,*S*)-α-methoxy-α-(trifluoromethyl)phenylacetyl esters of **4** (below), the DtcycB-catalyzed reaction was performed several times.

**Structural analysis of the diterpene products:** The structures of the diterpene products were analyzed by ^1^H NMR spectroscopy, ^13^C NMR spectroscopy, HMBC, HSQC, and COSY (600 MHz, ECA-600; JEOL, Tokyo, Japan). High-resolution Triple TOF 5600 MS apparatus (AB SCIEX, Tokyo, Japan) was used to determine the molecular formulae of the reaction products. MS analysis was performed by using electrospray ionization in the positive-ion mode. The optical rotations were recorded with a DIP-1000 polarimeter (JASCO, Tokyo, Japan).

**Preparation of (*R***,***S*****)-α-methoxy-α-(trifluoromethyl)phenylacetyl esters of 4:** Either (*S*)-α-methyl-α-trifluoromethylphenylacetyl (MTPA) chloride (10 μL) or (*R*)-MTPA chloride (10 μL) and 4-dimethylaminopyridine (1 mg) were added to a stirred solution of **4** (8 mg) in dry C_5_H_5_N (150 μL). The mixture was stirred at room temperature for three days, diluted with HCl (5 mL, 1 M), and extracted with ethyl acetate (3×5 mL). The combined extracts were successively washed with H_2_O, NaHCO_3_ (aq), and brine, dried with MgSO_4_, and concentrated. The residual oil was purified by preparative HPLC with a PEGASIL ODS column (20×250 mm) and isocratic elution (methanol (95 %), 8 mL min^−1^). The absorption of the eluate was monitored at 210 nm. (*S*)-MTPA ester (2 mg) and (*R*)-MTPA ester (2 mg) were obtained as colorless oils.

**Steady-state kinetic parameters:** Spectrophotometric assays (coupled system) with a pyrophosphate reagent (Sigma–Aldrich) were employed for steady-state kinetic studies of DtcycA and DtcycB, because both enzymes form a pyrophosphate anion coproduct during catalysis. The cyclase activity was measured in a total volume of 800 μL containing MgCl_2_ (10 mm), the pyrophosphate reagent (266 μL), and GGDP (10, 20, 40, 80, 100, 160, or 200 μm). After this reaction mixture (without enzyme) was incubated at 30 °C for 3 min, the reaction was initiated by adding DtcycA (532 μg) or DtcycB (466 μg). The enzyme-dependent oxidation of NADH was monitored by using a UV-1600PC spectrophotometer (Shimadzu, Kyoto, Japan) equipped with a CPS-240 A cell holder (Shimadzu) adjusted to 30 °C. Initial velocity was determined from the slope of a plot of NADH consumption against incubation time. The molar extinction coefficient (*ε*) of NADH is 6220 M^−1^ cm^−1^ at 340 nm. The steady-state kinetic parameters were calculated by using SigmaPlot 12.3 software (Systat Software, Point Richmond, CA).

**Accession numbers:** The nucleotide sequences of DtcycA and DtcycB have been deposited in the DDBJ/EMBL/GenBank nucleotide sequence database and assigned the accession numbers AB738084 and AB738085, respectively.

## Notes

As a service to our authors and readers, this journal provides supporting information supplied by the authors. Such materials are peer reviewed and may be re-organized for online delivery, but are not copy-edited or typeset. Technical support issues arising from supporting information (other than missing files) should be addressed to the authors.
