# Supplementary material for: Identification and Characterization of Bacterial Diterpene Cyclases that Synthesize the Cembrane Skeleton
Source: Chembiochem. 2013 Feb 6;14(3):316–21. doi: 10.1002/cbic.201200651 (PMC3790952; doi:10.1002/cbic.201200651)
Supplement: Supplementary file 1 [file cbic0014-0316-sd1.pdf]

CHEMBIOCHEM

Supporting Information

© Copyright Wiley-VCH Verlag GmbH & Co. KGaA, 69451 Weinheim, 2013

Identification and Characterization of Bacterial Diterpene Cyclases that Synthesize the Cembrane Skeleton

Ayuko Meguro, Takeo Tomita, Makoto Nishiyama, and Tomohisa Kuzuyama*[a]

cbic_201200651_sm_miscellaneous_information.pdf

Supporting Information

^{1}H-NMR, ^{13}C-NMR, and MS spectral data and optical rotations of the diterpene products.

The isopropylidene isomer of cembrene C (**1**) is a known natural product, but we were unable to directly compare the spectral data of **1** to those of the isopropylidene isomer of cembrene C because of the lack of spectral data for the isopropylidene isomer of cembrene C.[1] In contrast, the identity of the diterpene products **2** and **3** generated by the recombinant DtcycA and DtcycB enzymes was established by direct comparison of the ^{1}H NMR, ^{13}C NMR and $[\alpha]^{20}_D$ data with literature data.

(1*E*,5*E*,9*E*)-1,5,9-Trimethyl-12-(propan-2-ylidene)cyclotetradeca-1,5,9-triene (1), the isopropylidene isomer of cembrene C, was generated by the DtcycA-catalyzed reaction using GGDP as the substrate.

The molecular formula of **1** was deduced to be $C_{20}H_{32}$ using positive high-resolution mass spectrometry (*m/z* 273.2576 $[M+H]^+$, calcd. for $C_{20}H_{33}$, 273.2582). The ^1H NMR spectrum (Figure S8) of product **1** indicated the presence of three olefinic protons (δ 4.92, 4.99, and 5.02 ppm; each 1H, triplet, J = 6.8, 7.6, and 7.6 Hz), and three tertiary olefinic carbon signals (δ 124.1, 124.8, 126.3 ppm) and five quaternary olefinic carbon signals (δ 124.7, 131.2, 133.1, 133.7, and 135.0 ppm) were apparent in the ^{13}C NMR spectrum (Figure S9), indicating that compound **1** is a monocyclic compound with four double bonds in the ring. All methyl groups exhibited resonances that are typical of allylic methyl groups (^1H, δ 1.54, 1.57, 1.58, 1.63, and 1.65; each 3H, singlet; ^{13}C, δ 15.0, 15.3, 15.9, 20.5, and 20.7 ppm, respectively). The 14 remaining protons appeared in the region at δ 1.93 − 2.80 ppm (Table S1). The directly bonded carbon and hydrogen atoms were assigned based on the HSQC spectrum. Using an extensive NMR spectroscopic analysis, including COSY and HMBC experiments, we determined that the structure of **1** is (1*E*,5*E*,9*E*)-1,5,9-trimethyl-12-(propan-2-ylidene)cyclotetradeca-1,5,9-triene. Selected key HMBC and COSY correlations of **1** are shown below.

Table S1. ^1H- and ^{13}C-NMR spectral data for diterpene **1**.

		**1**		
#		δc	$δ_H$	HMBC
1	C=	133.7		
2	CH_2	39.0	2.11 (t, 6.2)	1, 3, 4, 14, 15
3	CH_2	24.9	2.16 (m)	2, 4
4	CH=	126.3	4.92 (t, 6.8)	3, 6, 16
5	C=	133.1		
6	CH_2	40.0	2.00 (t, 5.8)	4, 5, 7, 8, 16
7	CH_2	23.8	2.05 (m)	6, 8
8	CH=	124.1	4.99 (t, 7.6)	6, 7, 10, 17
9	C=	135.0		
10	CH_2	37.4	1.93 (t, 7.6)	8, 9, 11, 12, 17
11	CH_2	31.4	2.16 (t, 7.6)	9, 10, 12
12	C=	131.2		
13	CH_2	30.5	2.80 (d, 7.6)	1, 11, 12
14	CH=	124.8	5.02 (t, 7.6)	2, 13
15	CH_3	15.9	1.58 (s)	1, 2, 14
16	CH_3	15.0	1.54 (s)	4, 5, 6
17	CH_3	15.3	1.57 (s)	8, 9, 10
18	C=	124.7		
19	CH_3	20.5	1.63 (s)	12, 18, 20
20	CH_3	20.7	1.65 (s)	12, 18

The data were recorded using $CDCl_3$.

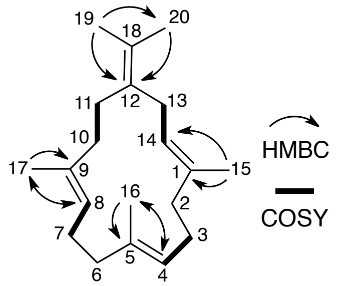

2-((S,3*E*,7*E*,11*E*)-4,8,12-Trimethylcyclotetradeca-3,7,11-trien-1-yl)propan-2-ol (2) ((*R*)-nephthenol) was generated by the DtcycA- and DtcycB-catalyzed reactions using GGDP as the substrate.

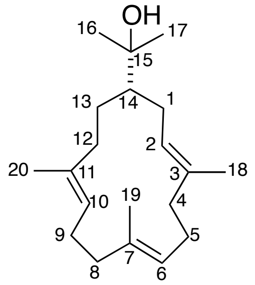

Table S2. ^{1}H- and ^{13}C-NMR spectral data for diterpene **2** and (*R*)-nephthenol.

	**2**[a]		(*R*)-nephthenol[b]
#	δ_C	δ_H	δ_C
1	28.5	2.1 (m), 1.88 (m)	28.42
2	126.0	5.09 (t, 7.2)	125.93
3	133.4		133.33
4	38.9	2.09 (m)	38.81
5	24.7	2.18 (m), 2.13 (m)	24.64
6	125.8	4.93 (t, 6.4)	125.74
7	133.1		133.02
8	39.5	2.00 (m, 6.2)	39.39
9	24.0	2.09 (m)	23.99
10	125.0	4.99 (t, 6.0)	124.96
11	134.1		134.02
12	37.7	2.06 (m), 2.01 (m)	37.69
13	28.3	1.63 (m), 1.25 (m)	28.26
14	48.5	1.31 (m)	48.44
15	74.1		73.93
16	27.7	1.19 (s)	27.64
17	27.5	1.19 (s)	27.48
18	15.6	1.54 (s)	15.54
19	15.3	1.56 (s)	15.28
20	15.6	1.54 (s)	15.53

[a]The data were recorded using $CDCl_3$. [b]The data were recorded using $CDCl_3$.[2]

Specific optical rotation of **2**, $[\alpha]^{20}_D = -31°$ (c = 0.61, $CHCl_3$).
Specific optical rotation of (*R*)-nephthenol, $[\alpha]^{20}_D = -39.6°$ (c = 1.11, $CHCl_3$).[2]

(S,1*E*,5*E*,9*E*)-1,5,9-Trimethyl-12-(prop-1-en-2-yl)cyclotetradeca-1,5,9-triene (3) ((*R*)-cembrene A) was generated by the DtcycB-catalyzed reaction using GGDP as the substrate.

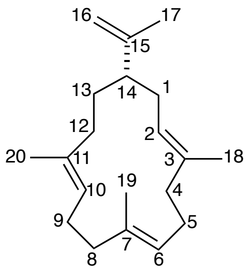

Table S3. ^{1}H- and ^{13}C-NMR spectral data for diterpene **3** and (*R*)-cembrane A.

	**3**[a]		(*R*)-cembrene A[b]
#	δ_C	δ_H	δ_C
1	32.5	1.98 (m)	32.43
2	121.9	5.05 (t, 6.1)	121.87
3	134.0		133.91
4	39.0	2.02 (m)	38.94
5	24.9	2.15 (m)	24.89
6	124.1	5.18 (t, 7.2)	124.07
7	134.9		134.79
8	39.5	2.06 (m)	39.41
9	23.8	2.12 (m)	23.76
10	126.0	4.97 (t, 6.6)	125.90
11	133.5		133.43
12	34.0	2.12 (m)	33.99
13	28.2	1.94 (m)	28.22
14	46.1	2.02 (m)	45.98
15	149.4		149.29
16	110.2	4.70 (s), 4.63 (s)	110.10
17	19.4	1.65 (s)	19.31
18	18.1	1.55 (s)	17.99
19	15.4	1.58 (s)	15.25
20	15.6	1.56 (s)	15.48

[a]The data were recorded using $CDCl_3$. [b]The data were recorded using $CDCl_3$.[2]

Specific optical rotation of **3**, $[\alpha]^{20}_D = -2.8°$ (c = 0.58, $CHCl_3$).
Specific optical rotation of (*R*)-cembrene A, $[\alpha]^{20}_D = -12°$ (c = 0.85, $CHCl_3$).[2]

LEGENDS FOR SUPPLEMENTARY FIGURES

Figure S1. Biosynthesis of the diterpenes isolated from actinomycetes. GGDP synthase catalyzes the condensation of one molecule of DMAPP and three molecules of IPP to yield GGDP. Diterpene cyclases catalyze the cyclization of GGDP to produce various diterpenes. Cyclooctat-9-en-7-ol synthase (CotB2) is a class I terpene synthase.[3] Terpentedienyl diphosphate synthase (Cyc1),[4] [5] *ent*-copalyl diphosphate synthase (SsCPS),[6] [7] and halimadienyl diphosphate synthase (Rv3377c)[8] are class II terpene cyclases. Terpentedienyl diphosphate is converted into terpentetriene by a class I terpene synthase Cyc2,[4] [5] *ent*-copalyl diphosphate is converted into (-)-pimara-9(11),15-diene by a class I terpene synthase ORF3,[6] [7] and halimadienyl diphosphate is converted into tuberculosinol and isotuberculosinol by a class I terpene synthase Rv3378c.[9] A pyrophosphate group remains in each reaction product formed by the class II enzymes, whereas a pyrophosphate group is missing in the reaction product formed by the class I enzyme. IPP, isopentenyl diphosphate; DMAPP, dimethylallyl diphosphate; GGDP, geranylgeranyl diphosphate.

Figure S2. Biosynthetic gene clusters involved in the production of diterpenes by *Streptomyces* strains. In each gene cluster, the terpene cyclase gene is located in a region flanking a GGDP synthase gene. *A*, terpentecin-producing *Kitasatospora griseola*[4] [5]; *B*, viguiepinol-producing *Streptomyces* sp. KO-3988[6] [7]; *C*, cyclooctain-producing *Streptomyces melanosporofaciens* MI614-43F2.[3]

Figure S3. Gene clusters containing the diterpene cyclases DtcycA and DtcycB. Only genes with the same direction as those of the diterpene cyclases are shown. Each GGDP synthase is located immediately upstream of each diterpene cyclase. The sequences of both GGDP synthase as partial sequences. Hypothetical proteins, which may be involved in modification of the diterpene products, are located downstream of each diterpene cyclase.

Figure S4. Alignment of two novel diterpene cyclases DtcycA and DtcycB with CotB2, Cyc2, and ORF3. The NSE/DTE motif that is conserved among the five sequences is underlined. Accession numbers: DtcycA, AB738084; DtcycB, AB738085; CotB2, AB448947; Cyc2, AB048795; ORF3, AB183750.

Figure S5. Characterization of the DtcycA enzyme. *A*, SDS-PAGE analysis of the recombinant DtcycA protein. *B*, Molecular weight determined by gel filtration (*left*) and calculation (*right*). *C*, Michaelis-Menten plot of the DtcycA-catalyzed reaction using various concentrations of GGDP (0.01−0.2 mM). The K_m and k_{cat} values were determined using a hypobolic fit of the data with SigmaPlot.

Figure S6. Characterization of the DtcycB enzyme. *A*, SDS-PAGE analysis of the recombinant DtcycB protein. *B*, Molecular weight determined by gel filtration (*left*) and calculation (*right*). *C*, Michaelis-Menten plot of the DtcycB-catalyzed reaction using various concentrations of GGDP (0.01−0.2 mM). The K_m and k_{cat} values were determined using a hypobolic fit of the data with SigmaPlot.

Figure S7. MS spectra of the diterpene products **1**, **2**, **3**, and **4**.

Figure S8. ^{1}H NMR ($CDCl_3$) spectrum for **1**.

Figure S9. ^{13}C NMR ($CDCl_3$) spectrum for **1**.

Figure S10. COSY spectrum for **1**.

Figure S11. HSQC spectrum for **1**.

Figure S12. HMBC spectrum for **1**.

Figure S13. ^{1}H NMR ($CDCl_3$) spectrum for **4**.

Figure S14. ^{13}C NMR ($CDCl_3$) spectrum for **4**.

Figure S15. HSQC spectrum for **4**.

Figure S16. COSY spectrum for **4**.

Figure S17. HMBC spectrum for **4**.

Figure S18. ^{1}H NMR spectral data of the (*R*,*S*)-MTPA esters of **4**. The values of both δ(*R*) and δ(*S*) for the (*R*,*S*)-MTPA derivatives of **4** are presented in ppm in each structure.

REFERENCES.

[1] M. Kobayashi, K. Osabe, *Chem. Pharm. Bull.* **1989**, *37*, 631-636.

[2] R. Schwabe, I. Farkas, H. Pfander, *Helv. Chim. Acta* **1988**, 71, 292-297.

[3] S.-Y. Kim, P. Zhao, M. Igarashi, R. Sawa, T. Tomita, M. Nishiyama, T. Kuzuyama, *Chem. Biol.* **2009**, *16*, 736-743.

[4] T. Dairi, Y. Hamano, T. Kuzuyama, N. Itoh, K. Furihata, H. Seto, *J. Bacteriol.* **2001**, *183*, 6085-6094.

[5] Y. Hamano, T. Kuzuyama, N. Itoh, K. Furihata, H. Seto, T. Dairi, *J. Biol. Chem.* **2002**, *277*, 37098-37104.

[6] T. Kawasaki, T. Kuzuyama, Y. Kuwamori, N. Matsuura, N. Itoh, K. Furihata, H. Seto, T. Dairi, *J. Antibiot.* **2004**, *57*, 739-747.

[7] T. Kawasaki, Y. Hayashi, T. Kuzuyama, K. Furihata, N. Itoh, H. Seto, T. Dairi, *J. Bacteriol.* **2006**, *188*, 1236-1244.

[8] C. Nakano, T. Okamura, T. Sato, T. Dairi, T. Hoshino, *Chem. Comm.* **2005**, 1016-1018.

[9] C. Nakano, T. Ootsuka, K. Takayama, T. Mitsui, T. Sato, T. Hoshino, *Biosci. Biotechnol. Biochem*. **2011**, *75*, 75-81.

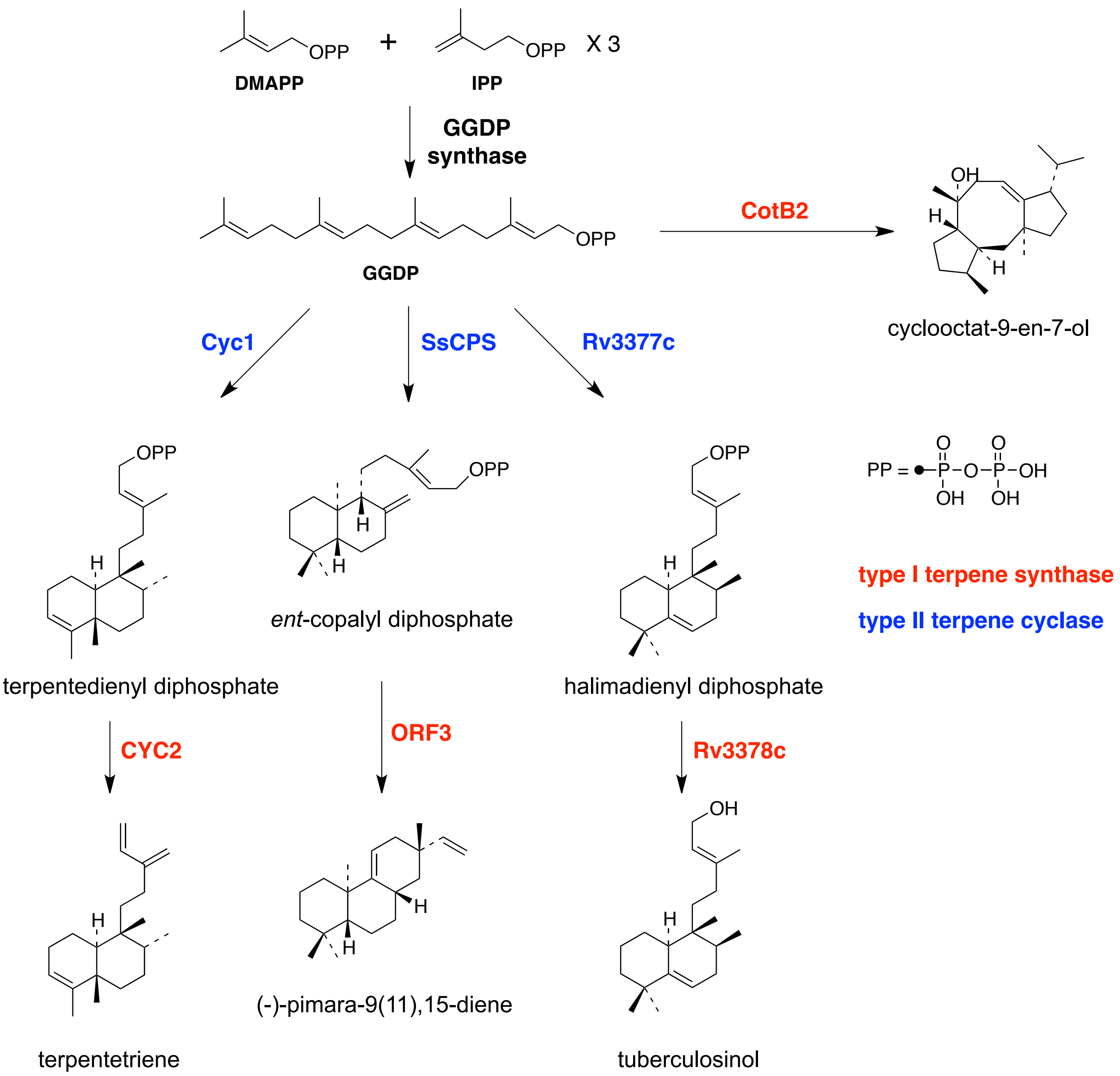
OPP
DMAPP
+
OPP
X 3
IPP
GGDP
synthase
OPP
GGDP
CotB2
OH
H
H
cyclooctat-9-en-7-ol
Cyc1
SsCPS
Rv3377c
OPP
H
terpentedienyl diphosphate
OPP
H
H
ent-copalyl diphosphate
OPP
H
halimadienyl diphosphate
PP = •–P–O–P–OH
O
O
OH
OH
type I terpene synthase
type II terpene cyclase
CYC2
ORF3
Rv3378c
H
terpentetriene
H
H
(-)-pimara-9(11),15-diene
OH
H
tuberculosinol

Figure S1

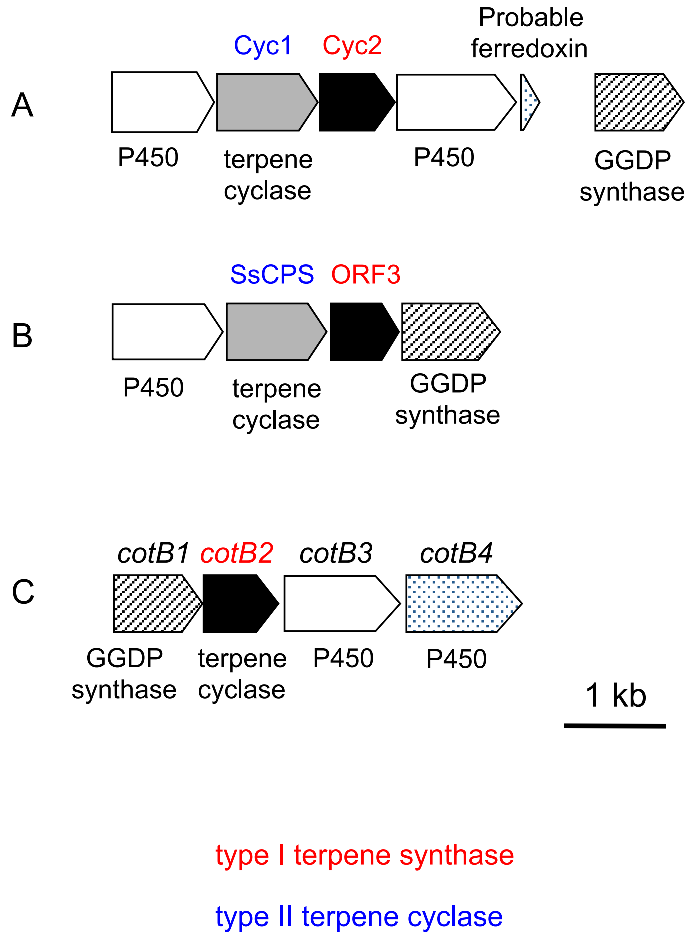
Probable
ferredoxin
Cyc1
Cyc2
A
P450
terpene
cyclase
P450
GGDP
synthase
SsCPS
ORF3
B
P450
terpene
cyclase
GGDP
synthase
cotB1
cotB2
cotB3
cotB4
C
GGDP
synthase
terpene
cyclase
P450
P450
1 kb
type I terpene synthase
type II terpene cyclase

Figure S2

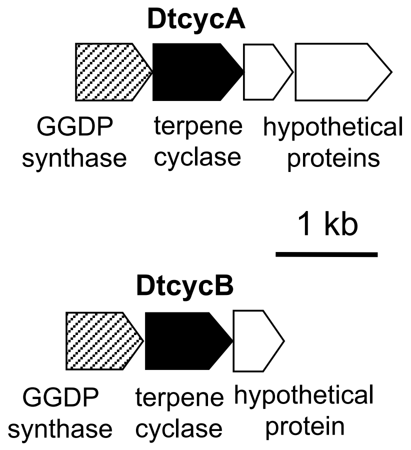
DtcycA
GGDP synthase
terpene cyclase
hypothetical proteins
1 kb
DtcycB
GGDP synthase
terpene cyclase
hypothetical protein

Figure S3

```
         1        10        20        30           40          50        60        70
DtcycA   MTDP.AVTPLAFSIPQLYCPFPTAIHPEVDT...LTRAGMDFMT..HHGFCNTEADRLVVANIDAGAIVARWYPNPDF.P
DtcycB   MDLPPAL..LSF.....YCP....IASEVSP...EHEAVAQEMYAWIHAMSLTSDNRQAKMLAQAGAG.....FNSYFTP
CotB2    MTTG.....LSTAGAQDIGR......SSVRPYLEECTRRFQEMFDRHVVTRPT.....KVELTDAELR.....EVIDDCN
Cyc2     .............................MPDAIEFEHEGRRNPNSAEAESAYSSIIAALDLQESDYAVISGHSRIVGAA
ORF3     ..................................................MRARHRVALKVLADLRSWAAEYPQVLEATP

                80        90       100        110       120            130          140
DtcycA   VDR..LQMVTDFLYLYFLIDDLRFEVINSDTG.LAGPIALFAQHLDLWEYPQAHRREE.....LDLFHQAIHD...LASR
DtcycB   RARGELARALSKYNVCAWIANGMVQEIR.DPG.TFG..AMAARWARIMEEP.ATCPAD.....GIPMDFALAD...AFSH
CotB2    AAVAPLGKTVSDE...RWISYVGVVLWSQSP...........RHIKDMEAFKAVCVLNCVTFVWDDMDPALHDFG.LFLP
Cyc2     ALVYPDADAETLLAASLWTACLIVNDDRWDYV.QED....GGRLAPGEWFDGVTEVVDT....WRTAGPRLPDP..FFEL
ORF3     IEALAISTAAISPWRGANELRLSAPDVRCGPTPLDDHVEQNVRSLDELDDLFGRCEAIVRGGDRDDGHPLLASLSGWQSA

              150            160           170            180           190           200
DtcycA   MAELTTPTKAA.....RMRRSING....WFLALLREIALFNDD.....HAVM...AEEYLPIRVVT....VASRLMIDVN
DtcycB   IRRTLSPVKWQ.....HFSAAQSH....WMHGLAWENCL.........HQVKGLTVHDYLSFRYVMSGCFAAAAFAYAVP
CotB2    QLR.KICEKYY.....GPEDAEVA....YEAARAFVTSDHMFRDSPIKAALCTTSPEQYFRFRVTDIG.VDFWMKMSYPI
Cyc2     VRTTMSRLDAA.....LGAEAADE....IGHEIKRAITAMKWEG.VWNEYTKKTSLATYLSFRRGYCTMDVQVVLDKWIN
ORF3     LERAPHYPKLAGLWGDRFAEALRGERYDWTAGLARDRGEGPSD............PQEYL.........TYAASSNAWIT

                210         220       230       240          250       260       270
DtcycA   GFICPAEVPGD..EWYSLKVQAAAEAAMSVCLYDNELYSAGKE...QWLKSRATAHDRRPRNLVALIQAQTGGSTEHALQ
DtcycB   ERHPSAE......EWAHPKVRAAADAAMMVDALDNDRYSYLKESLTEADKKTIFAALR.HEN.PAL......GREEVIVR
CotB2    YRHPEFT........EHAKTSLAARMTTRGLTIVNDFYSYDREVS...........LGQITNCFRLCD....VSDETAFK
Cyc2     GGRSFAALR......DDPVRRAIDDVVVRFGCLSNDYYSWGRE......KK...AVDK..SNAVRILMDHAGYDESTALA
ORF3     HFPRWATSDRDDLLDGLPVLDNALEAIEVAVRLSNDLATFERER...........AEPGQNN.......ILMYDTSPDWV

           280        290            300       310       320        330       340
DtcycA   EVAEYRNRTVC.LYLNLRSQ.....LEKTASPALLAYLSVLDGVISGNLDAHATS.SRYHNPDGHHPHAIAFTPLRTTDE
DtcycB   GVQL.RDRILT.LYLTLRGE.....LLCDASEGLRSYLTGLDLIIAGNLVFCADMGLRYGLPEGS.........VRTDAE
CotB2    EFFQAR......LDDMIEDI.....ECIKAFDQLT.QDVFLDL.IYGNFV.WTTSNKRYKTAVNDVNSRIQ.........
Cyc2     HVRDDCVQAITDLDCIEESIKRSGHLGSHAQELLD.YLACHRPLIYAAAT.WPTETNRYR....................
ORF3     HDELDRHSRKAQEQLDPLATAG....FPPAVELLR.LLDWSVTFYSGADFRGWGSDRDLTGPSGLPSDM...........

        350       360          370
DtcycA   CSARAHTPIAPP....IAWWWEQLDQ
DtcycB   PLDR..T.VAPPGIGAIDHWWAQAGA
CotB2    ..........................
Cyc2     ..........................
ORF3     ..........................
```

Figure S4

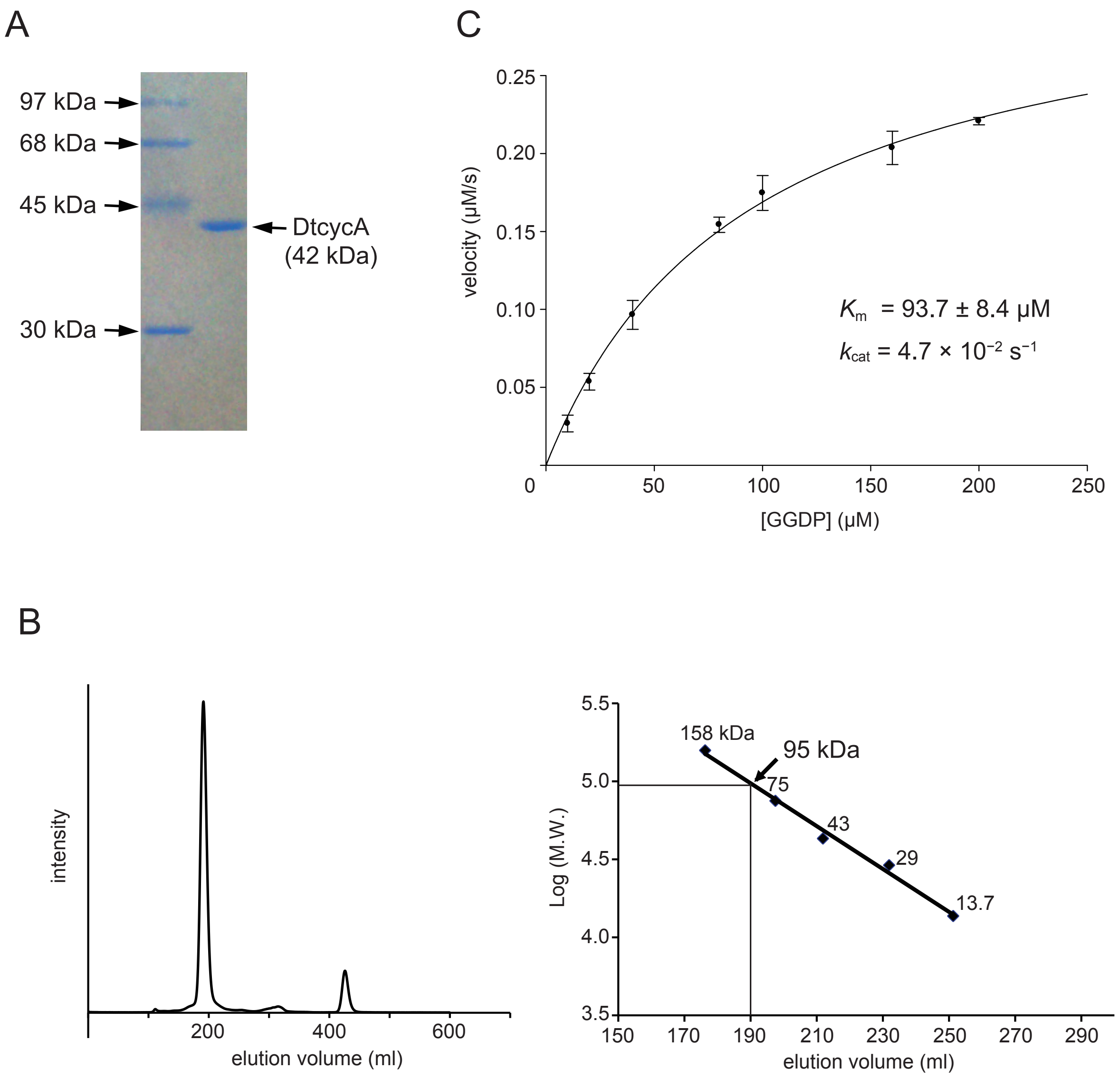
A
97 kDa
68 kDa
45 kDa
30 kDa
DtcycA
(42 kDa)
C
velocity (μM/s)
0.25
0.20
0.15
0.10
0.05
0
50
100
150
200
250
[GGDP] (μM)
K_m = 93.7 ± 8.4 μM
k_{cat} = 4.7 × 10^{-2} s^{-1}
B
intensity
200
400
600
elution volume (ml)
Log (M.W.)
5.5
5.0
4.5
4.0
3.5
158 kDa
95 kDa
75
43
29
13.7
150
170
190
210
230
250
270
290
elution volume (ml)

Figure S5

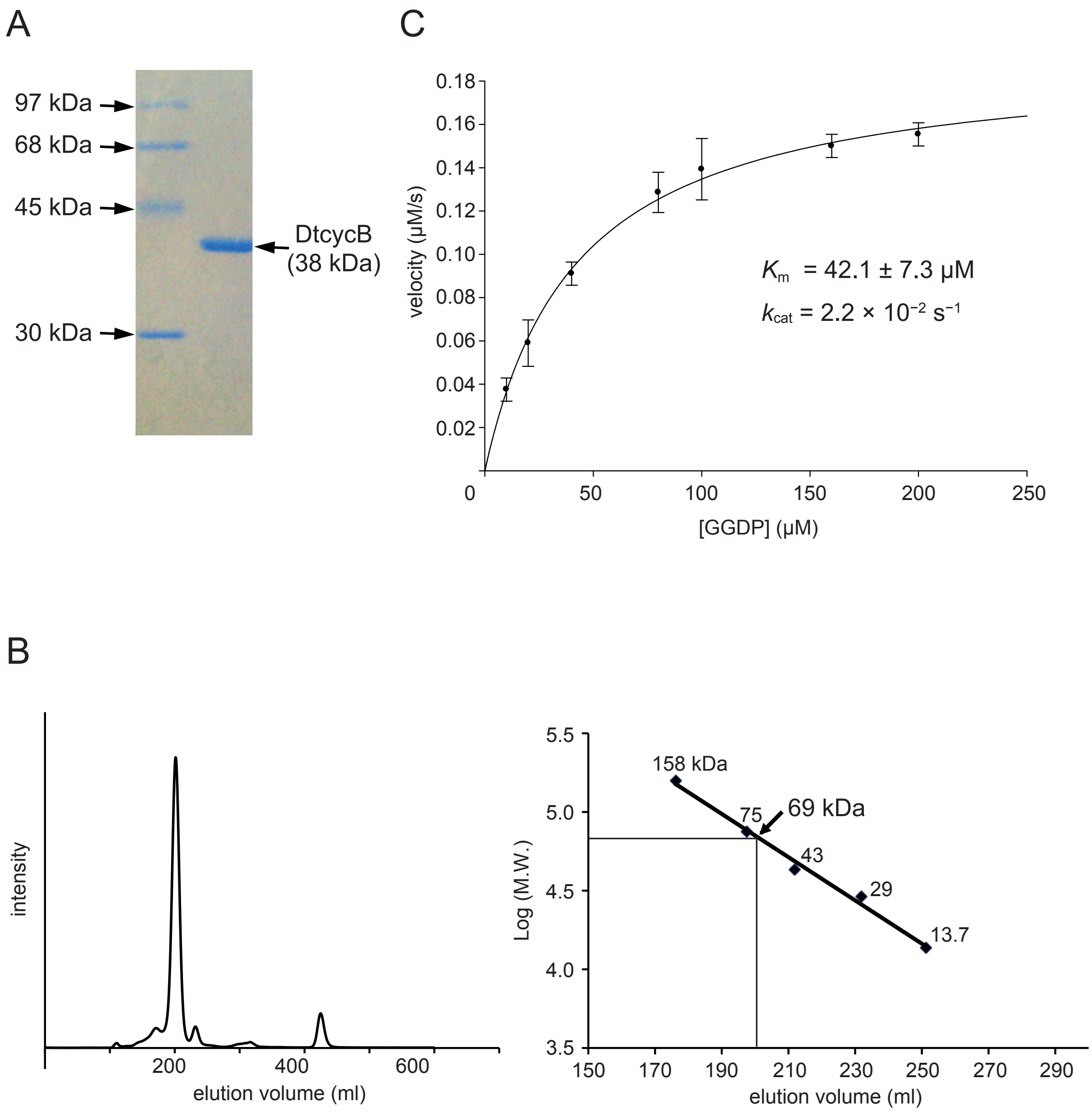
A
97 kDa
68 kDa
45 kDa
30 kDa
DtcycB
(38 kDa)
C
velocity (μM/s)
0.18
0.16
0.14
0.12
0.10
0.08
0.06
0.04
0.02
0
50
100
150
200
250
[GGDP] (μM)
K_m = 42.1 ± 7.3 μM
k_{cat} = 2.2 × 10^{-2} s^{-1}
B
intensity
200
400
600
elution volume (ml)
Log (M.W.)
5.5
5.0
4.5
4.0
3.5
158 kDa
75
69 kDa
43
29
13.7
150
170
190
210
230
250
270
290
elution volume (ml)

Figure S6

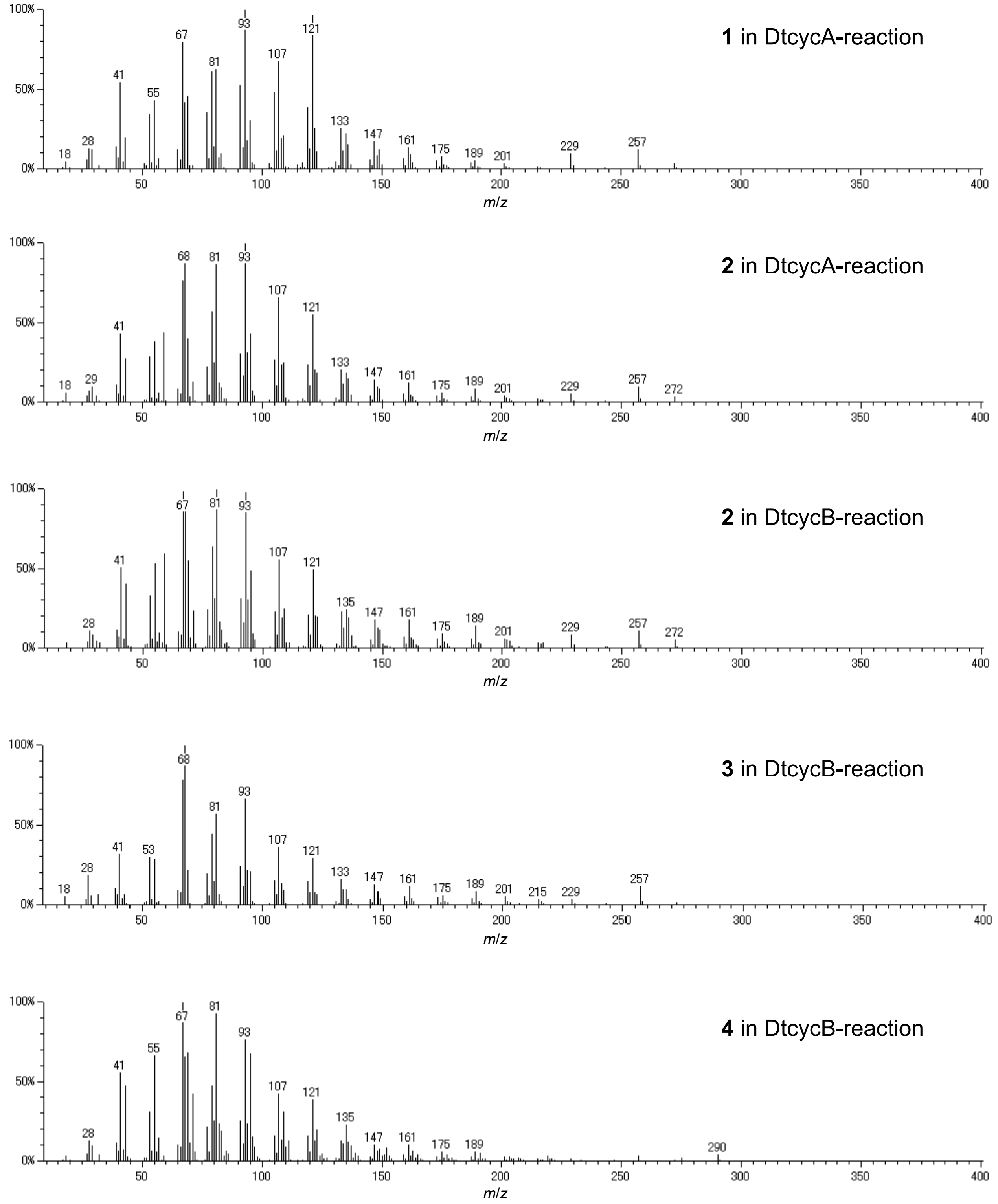

1 in DtcycA-reaction
18 28 41 55 67 81 93 107 121 133 147 161 175 189 201 229 257
2 in DtcycA-reaction
18 29 41 68 81 93 107 121 133 147 161 175 189 201 229 257 272
2 in DtcycB-reaction
28 41 67 81 93 107 121 135 147 161 175 189 201 229 257 272
3 in DtcycB-reaction
18 28 41 53 68 81 93 107 121 133 147 161 175 189 201 215 229 257
4 in DtcycB-reaction
28 41 55 67 81 93 107 121 135 147 161 175 189 290
100%
50%
0%
50 100 150 200 250 300 350 400
m/z

Figure S7

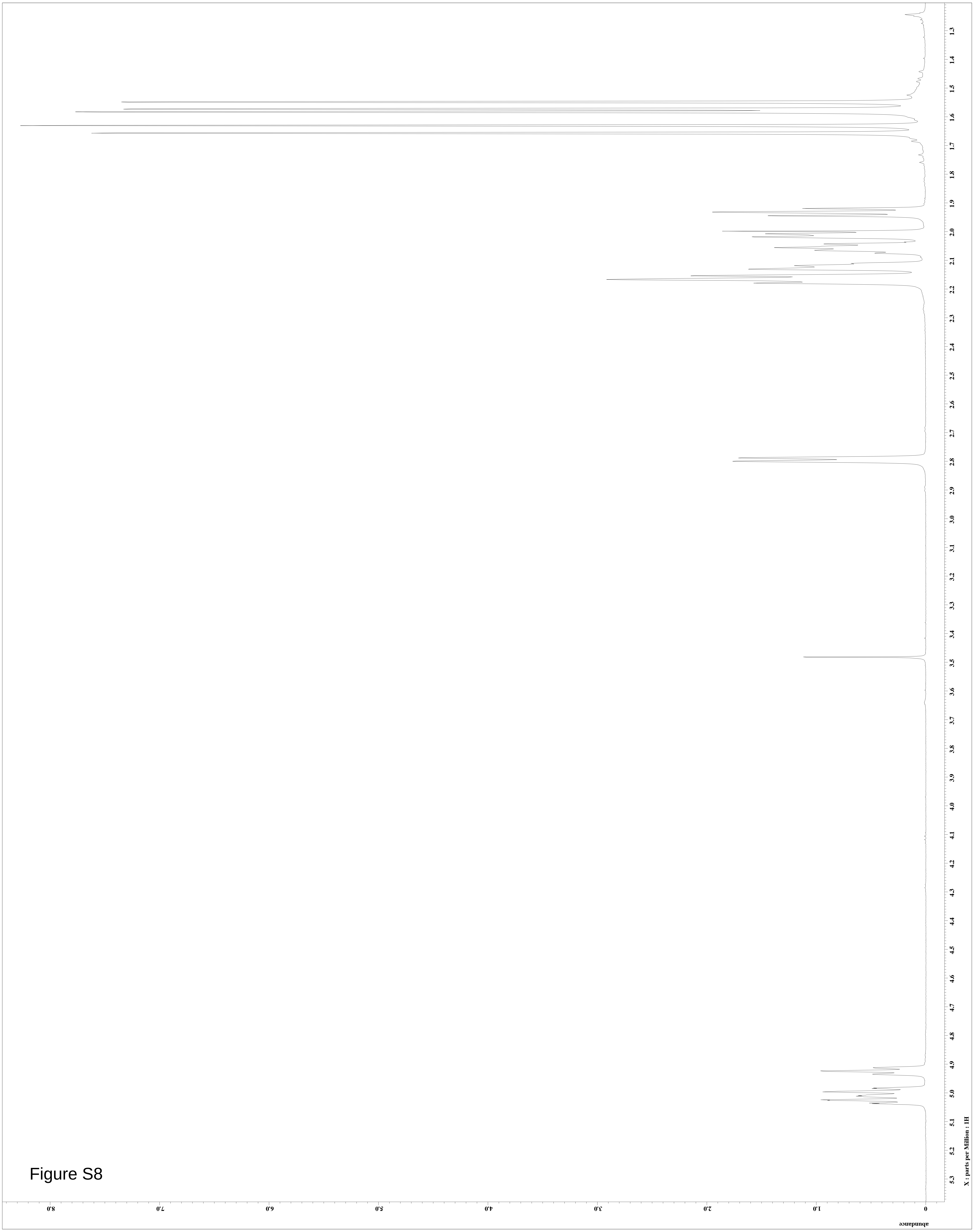

Figure S8

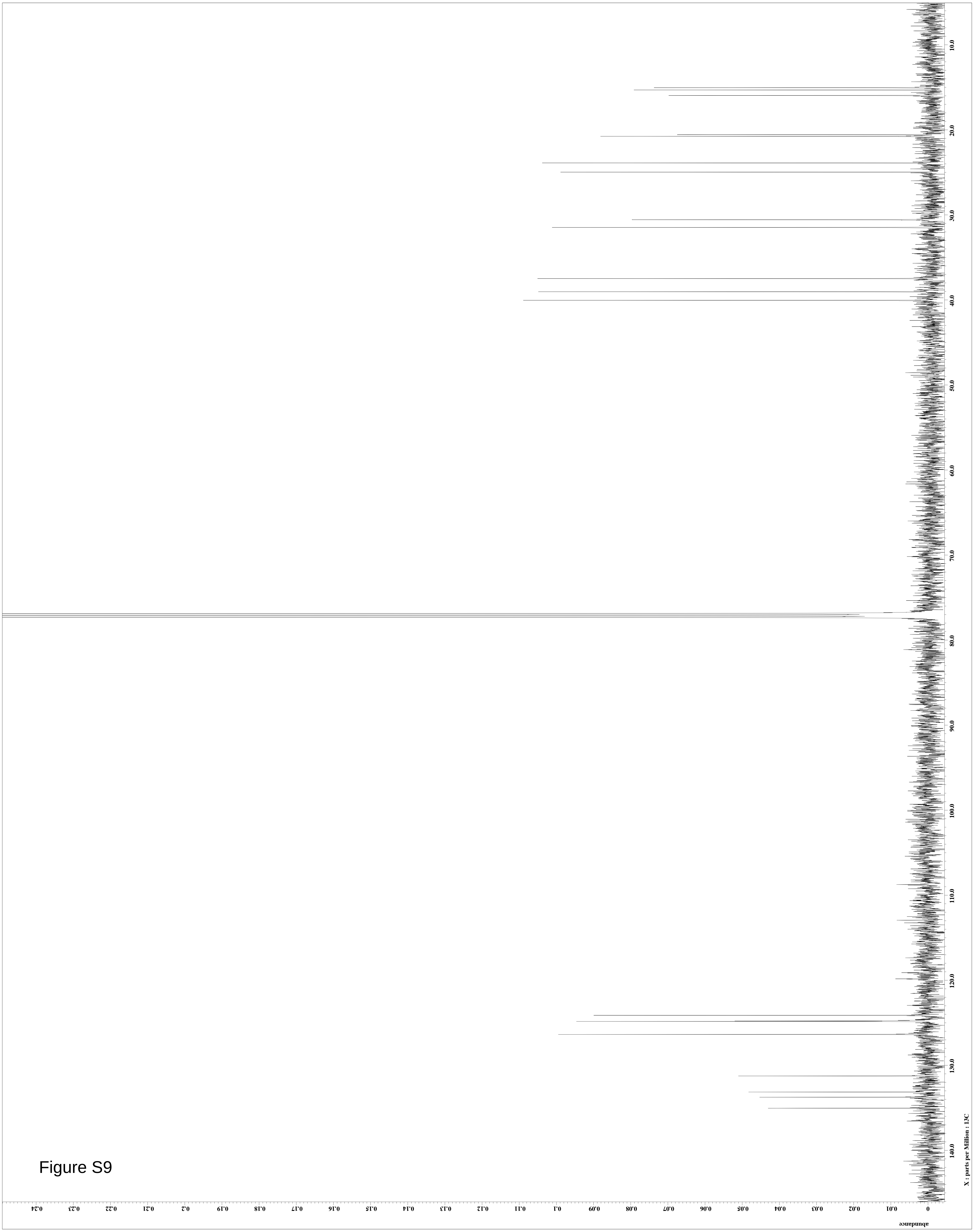

Figure S9

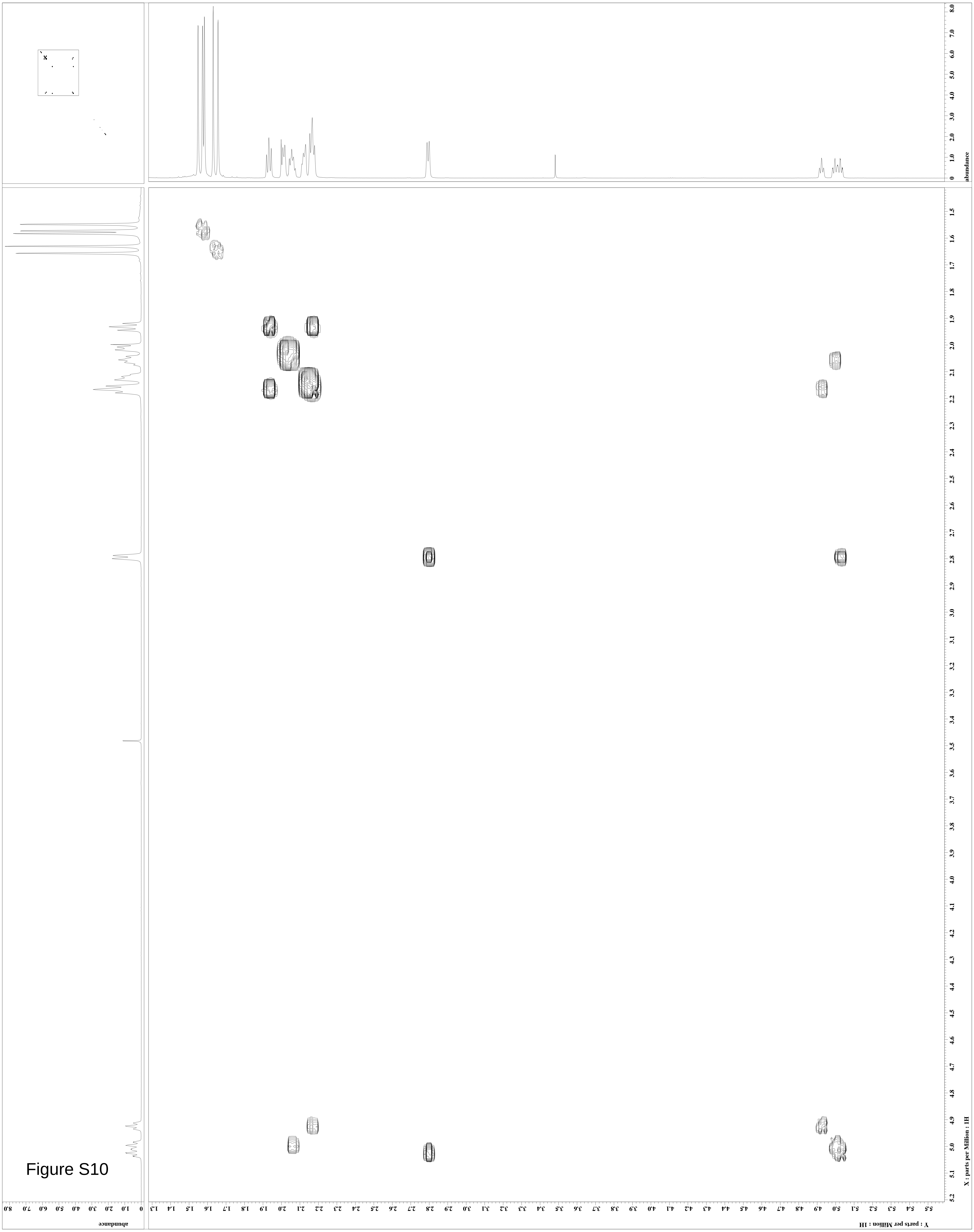

Figure S10

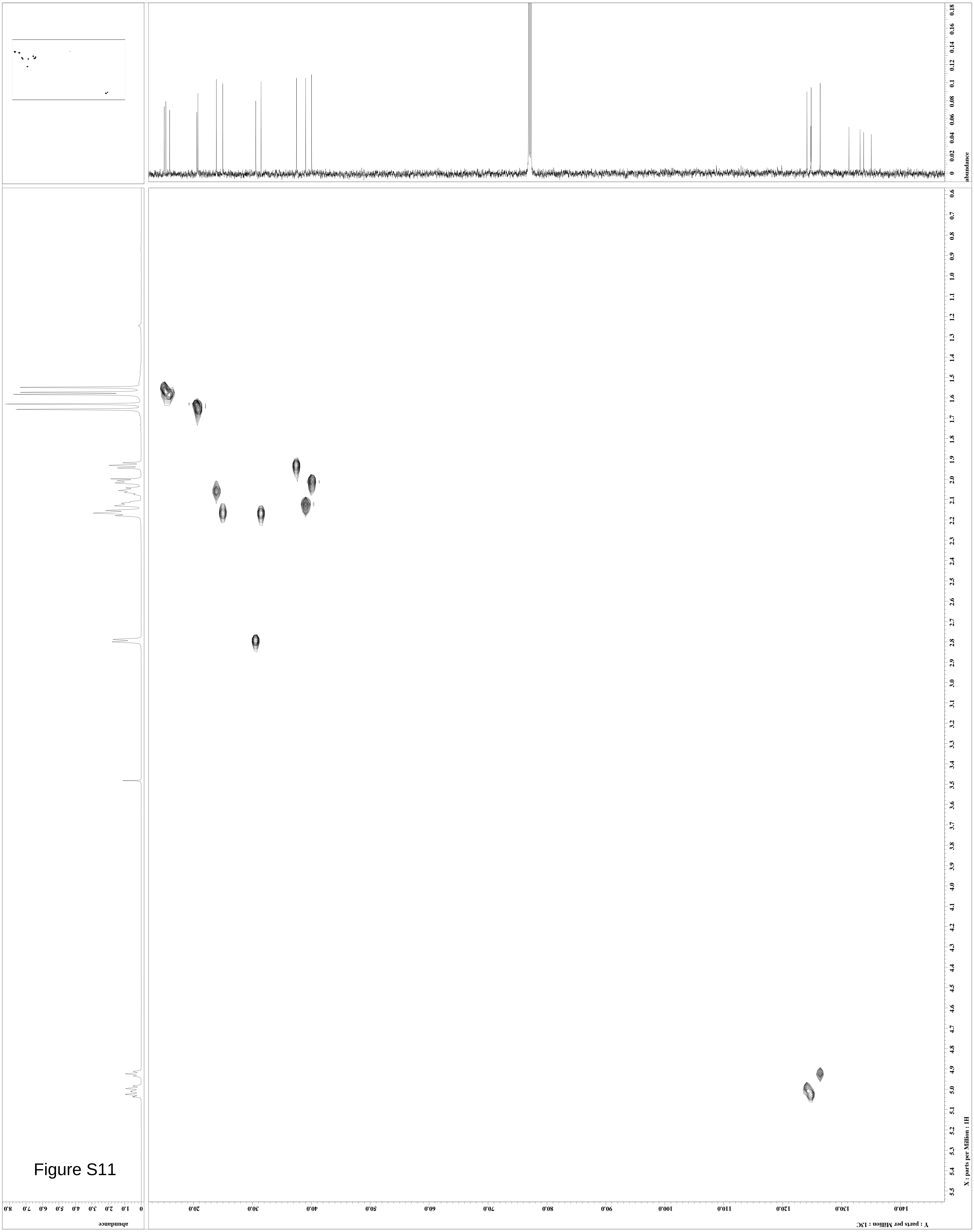

Figure S11

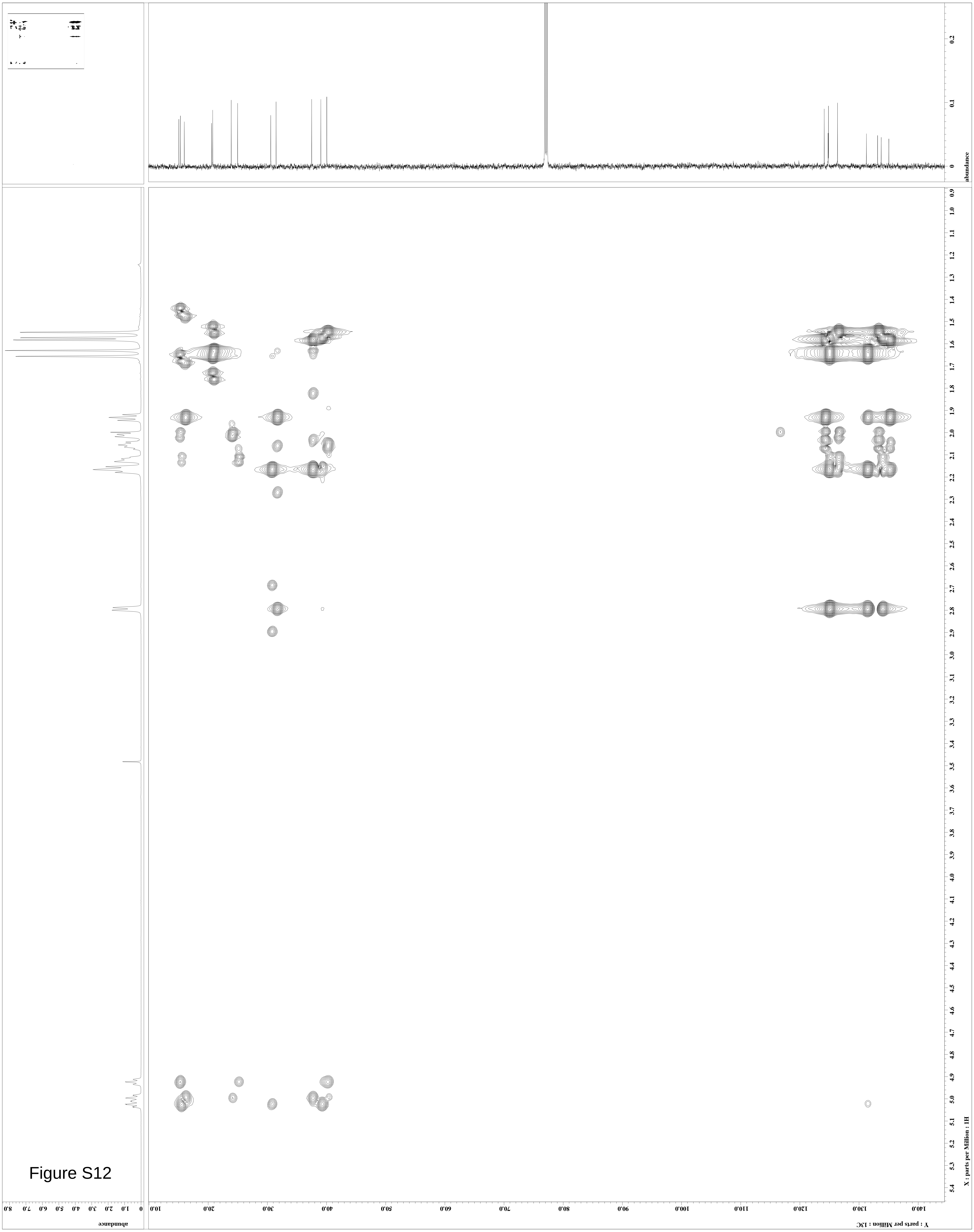

Figure S12

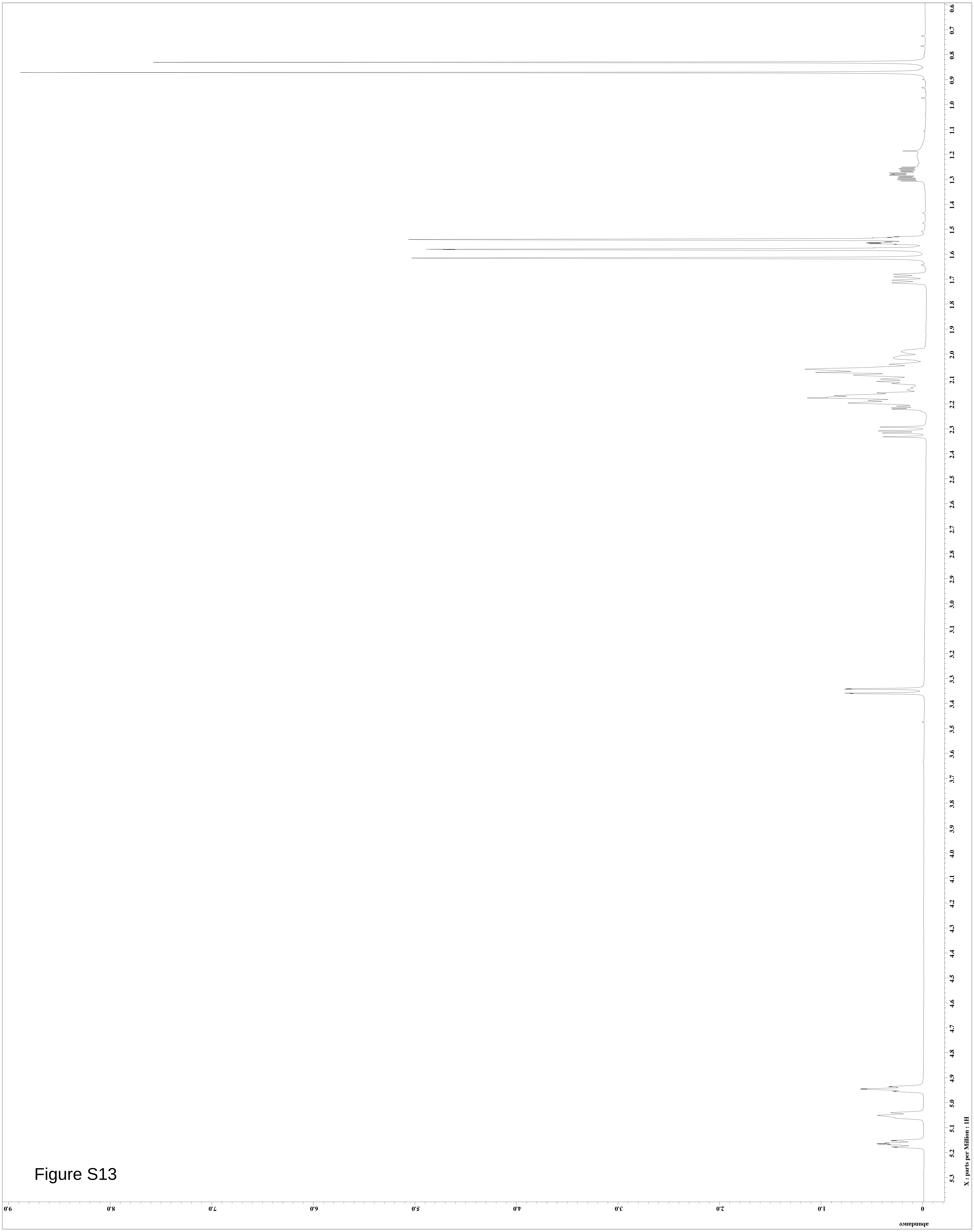

Figure S13

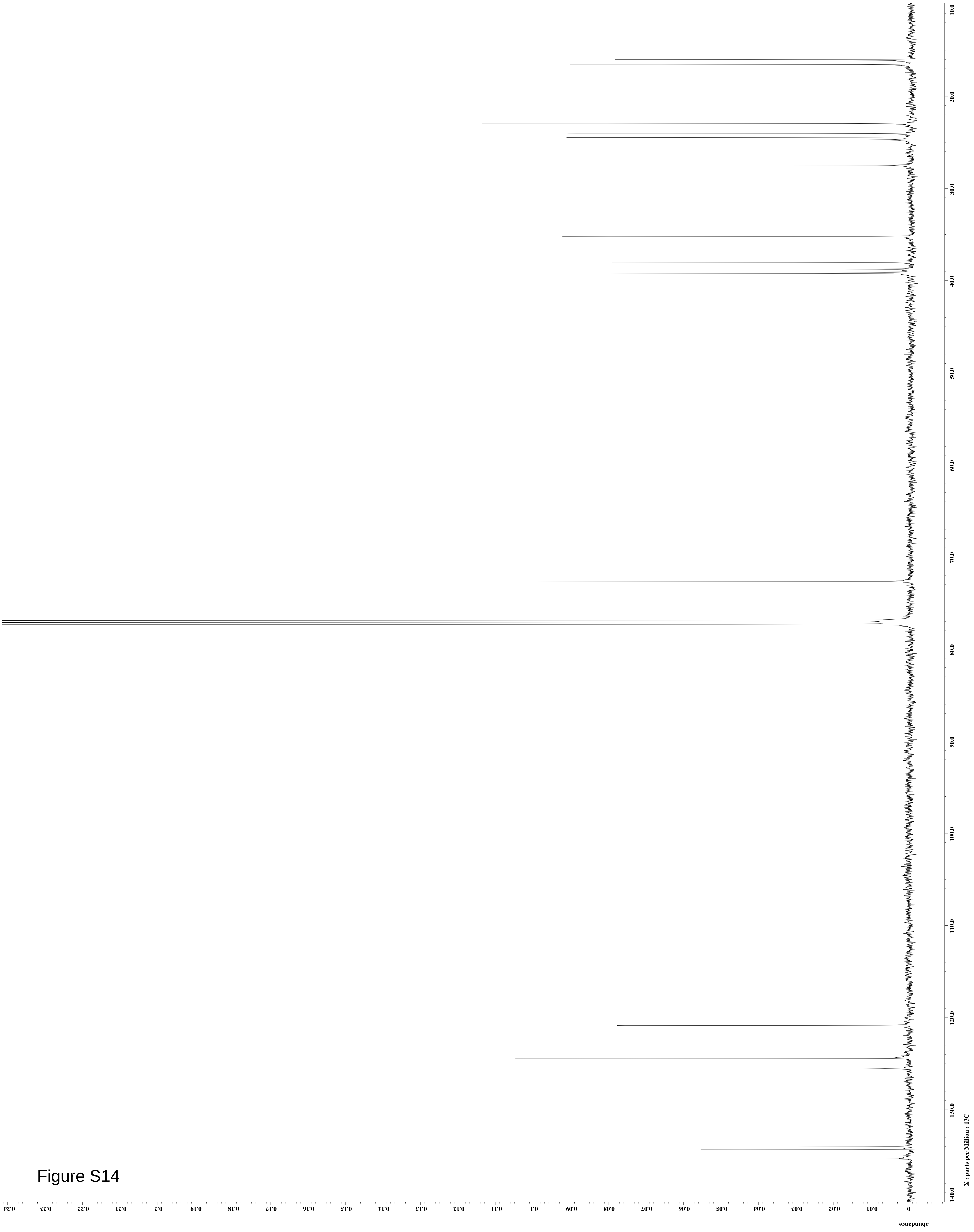

Figure S14

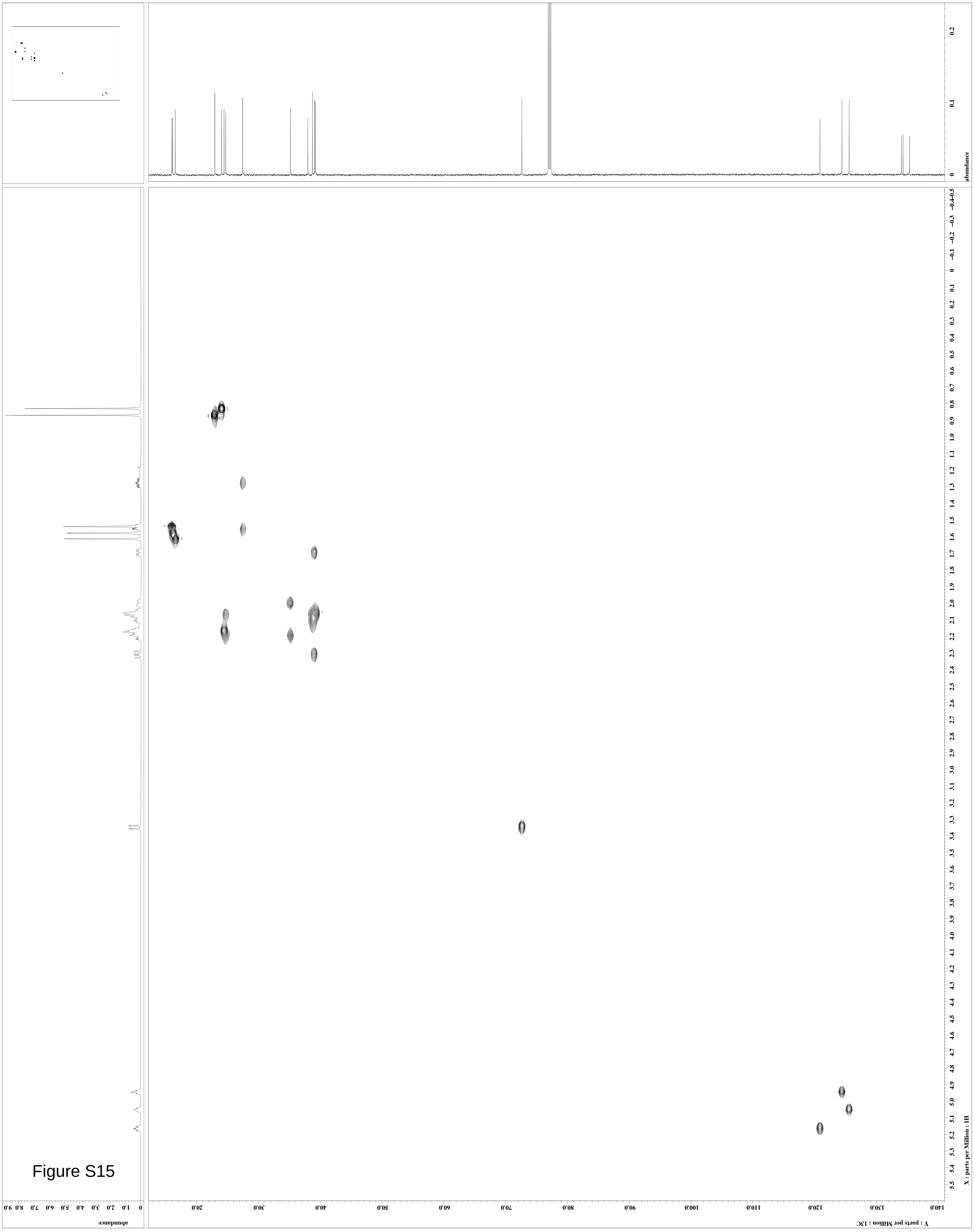

Figure S15

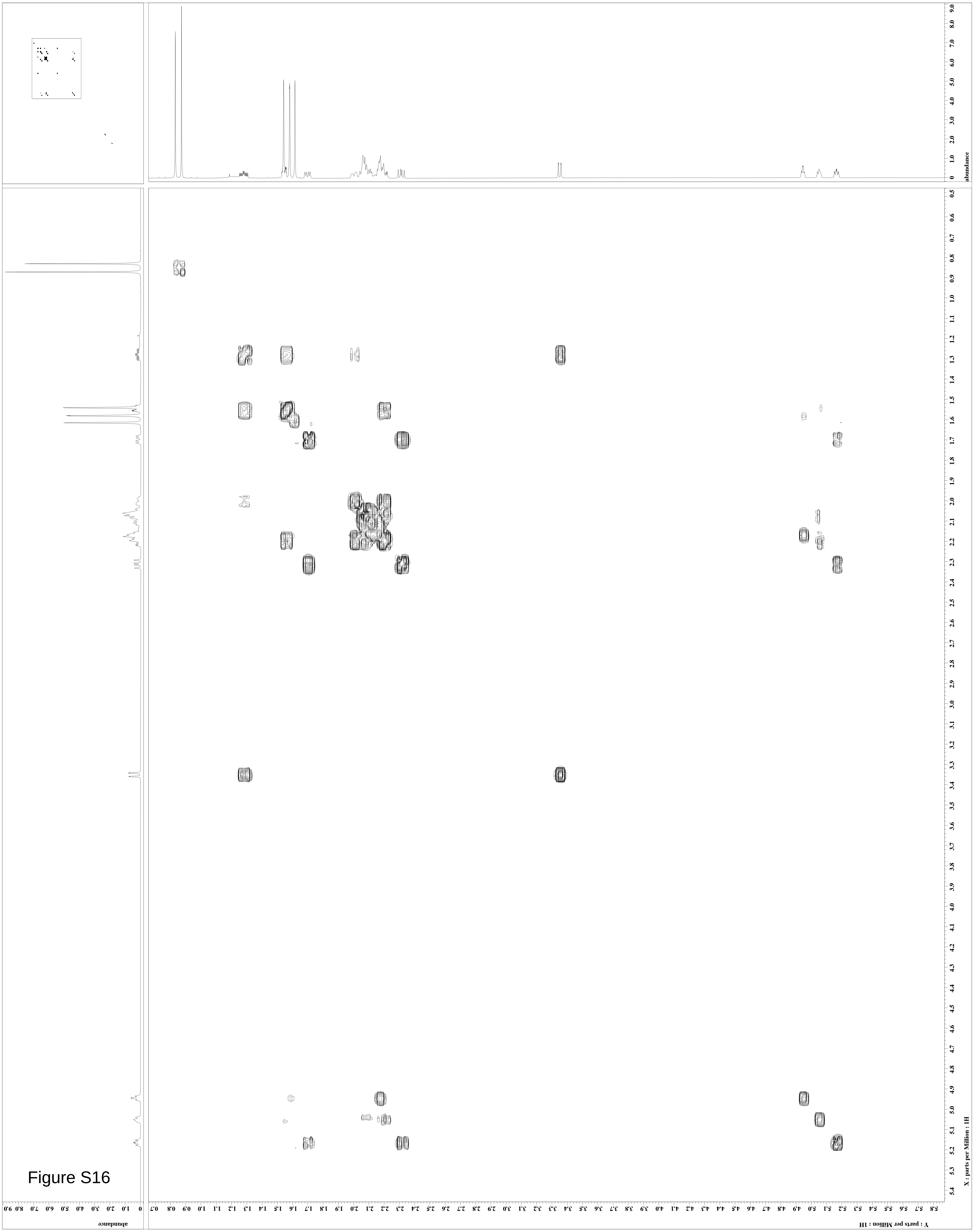

Figure S16

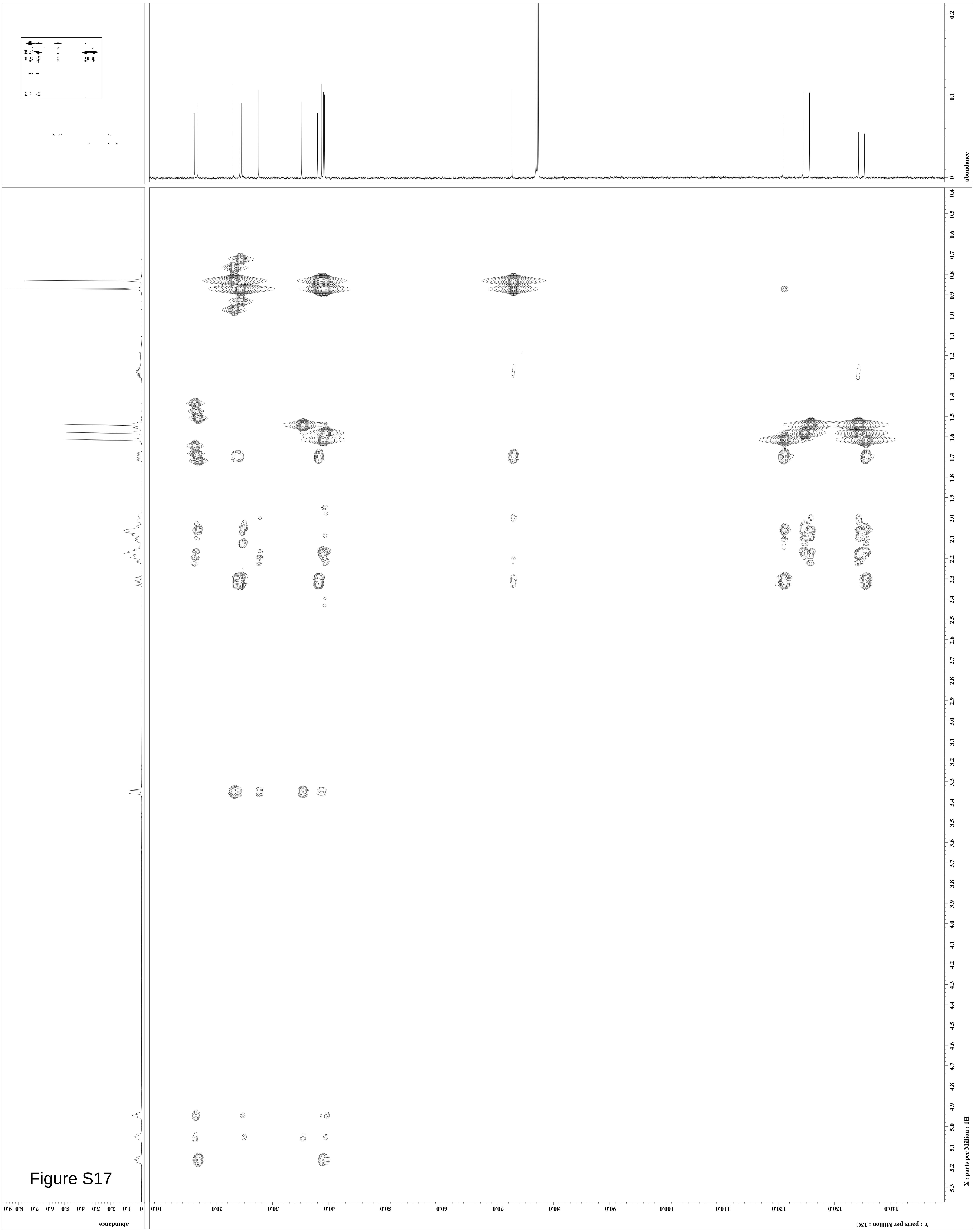
abundance
X : parts per Million : 1H
Y : parts per Million : 13C
abundance

Figure S17

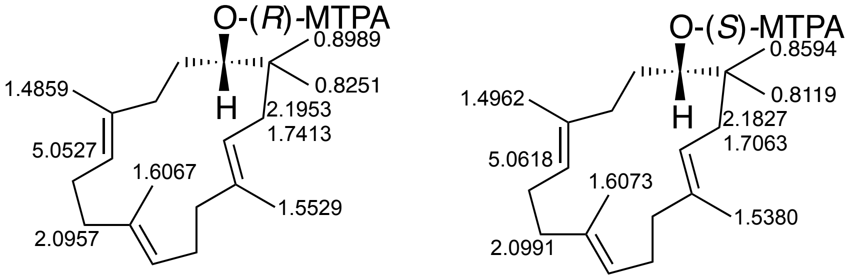
O-(*R*)-MTPA
0.8989
0.8251
1.4859
H
2.1953
1.7413
5.0527
1.6067
1.5529
2.0957
O-(*S*)-MTPA
0.8594
0.8119
1.4962
H
2.1827
1.7063
5.0618
1.6073
1.5380
2.0991

Figure S18
